# Computational modelling of meiotic entry and commitment

**DOI:** 10.1038/s41598-017-17478-9

**Published:** 2018-01-09

**Authors:** Tanvi Bhola, Orsolya Kapuy, P. K. Vinod

**Affiliations:** 1 Center for Computational Natural Sciences and Bioinformatics, International Institute of Information Technology, Hyderabad, 500032 India; 2 Semmelweis University, Department of Medical Chemistry, Molecular Biology and Pathobiochemistry, Budapest, Hungary

## Abstract

In response to developmental and environmental conditions, cells exit the mitotic cell cycle and enter the meiosis program to generate haploid gametes from diploid germ cells. Once cells decide to enter the meiosis program they become irreversibly committed to the completion of meiosis irrespective of the presence of cue signals. How meiotic entry and commitment occur due to the dynamics of the regulatory network is not well understood. Therefore, we constructed a mathematical model of the regulatory network that controls the transition from mitosis to meiosis in *Schizosaccharomyces pombe*. Upon nitrogen starvation, yeast cells exit mitosis and undergo conjugation and meiotic entry. The model includes the regulation of Mei2, an RNA binding protein required for conjugation and meiotic entry, by multiple feedback loops involving Pat1, a kinase that keeps cells in mitosis, and Ste11, a transcription activator required for the sexual differentiation. The model accounts for various experimental observations and demonstrates that the activation of Mei2 is bistable, which ensures the irreversible commitment to meiosis. Further, we show by integrating the meiosis-specific regulation with a cell cycle model, the dynamics of cell cycle exit, G1 arrest and entry into meiosis under nitrogen starvation.

## Introduction

The fission yeast *Schizosaccharomyces pombe* is an interesting model system to study the switch from proliferation to differentiation at the unicellular level. In the presence of nutrients, the fission yeast proliferates in the haploid state by elongation, mitotic cellular division, septation and separation (“fission”). In the absence of nutrients, especially nitrogen, cells of opposite mating type, h+ and h−, transiently arrest in G1, mate to form diploid zygotes (h+/h−), and undergo meiosis to form four haploid spores^1,2^. Nitrogen starvation induces G1 arrest after two accelerated cell divisions. Further, zygotes can also grow and divide (diploid mitosis) if they are transferred to nutrient rich conditions immediately after conjugation. Therefore, meiosis entry requires nitrogen starvation and the diploidization.

Different mutants of fission yeast that block or promote meiosis have been isolated^1,3^. In particular, *mei1*, *mei2*, *mei3* and *mei4* are required for meiosis with *mei2* required for the meiotic commitment. *mei2* encodes an RNA binding protein that is required for premeiotic synthesis and meiosis I^4,5^. *mei3* deletion increases the chance (from 1% to 80%) of forming diploid colonies when transferred to nutrient rich conditions^1^. Further, temperature-sensitive *pat1-114* (or *ran1-3*), triggers ‘ectopic meiosis’ irrespective of nutritional or the ploidy of cells suggesting that it is a suppressor of meiosis^2,6,7^. The *pat1-114* driven haploid meiosis is suppressed by *mei2* deletion. Mei2 acts downstream of Pat1 kinase to promote meiosis and is inhibited by Pat1-dependent phosphorylation, which decreases its stability^8,9^. On the other hand, Mei3 acts upstream of Pat1 and inhibits it by forming an inhibitory complex^10,11^.

**Figure 1:**
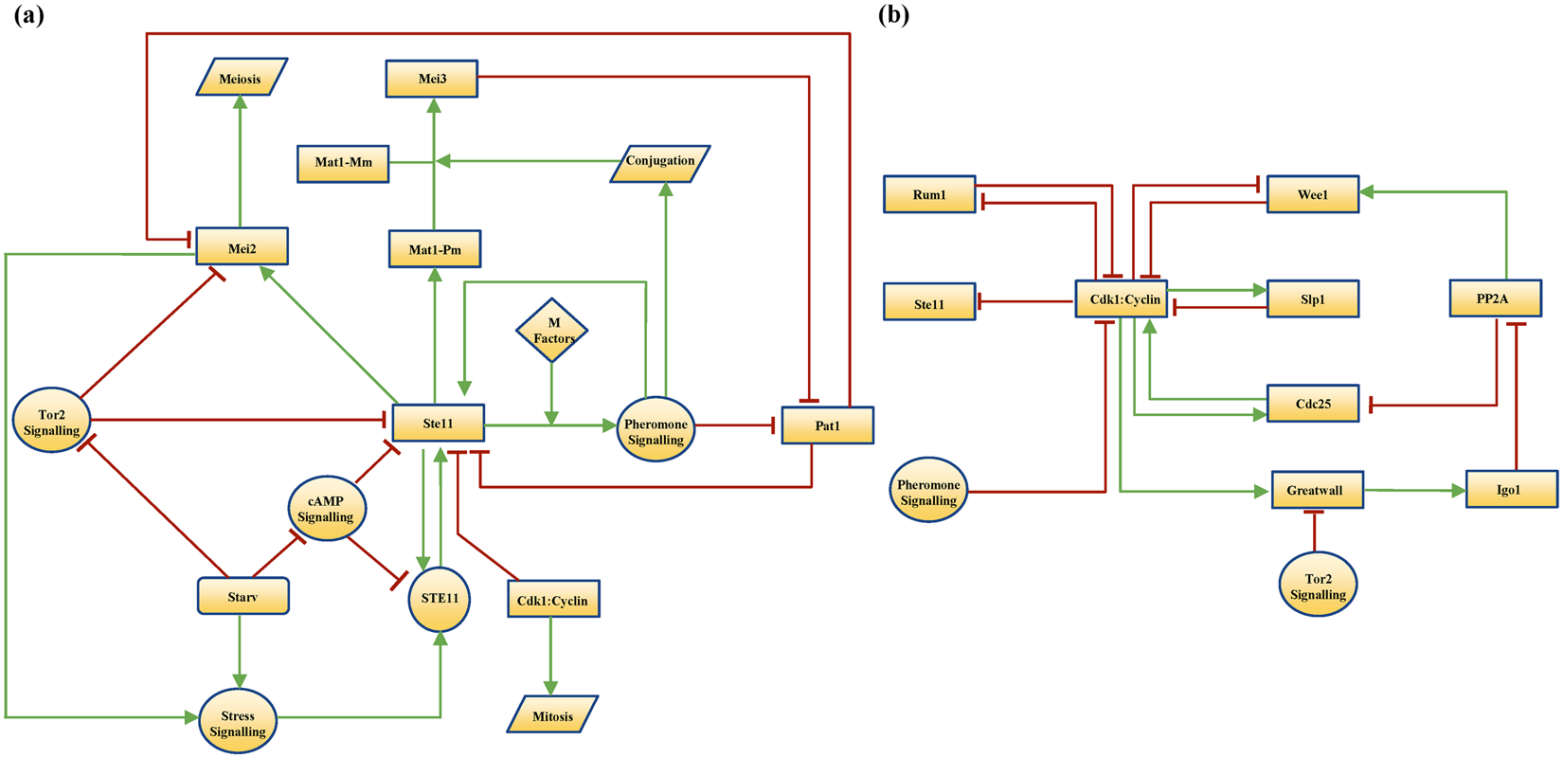
The wiring diagram of (**a**) meiosis entry and commitment network and (**b**) mitotic cell cycle network in the fission yeast.

The sequence of cellular differentiation, conjugation and meiosis, is initiated by the activation of an high-mobility group (HMG) family transcription factor Ste11, which controls the expression of itself, nitrogen starvation responsive genes, pheromone response genes, mating-type specific genes and *mei2* ^12,13^. Ste11 is subjected to both transcriptional and post transcriptional controls by nutrient, mating pheromone and stress responsive signalling pathways (Fig. 1a)^14–16^. Nitrogen starvation triggers the synthesis of Ste11 by decreasing the activity of cAMP-dependent protein kinase (PKA) and Tor2 kinase (TORC1). PKA controls the synthesis of Ste11 by suppressing the transcriptional activator Rst2^17,18^. Tor2-dependent phosphorylation of Mei2 promotes its degradation^19^. Mei2 in turn acts via stress-responsive Sty1 mitogen-activated protein kinase (MAPK) pathway to promote C-terminal domain of RNA polymerase II (Pol II CTD) phosphorylation by CTDK-I, which is essential for the synthesis of Ste11^20,21^. Sty1 is also shown to act via Atf1-Pcr1 to control Ste11 expression^22^. The nuclear-cytoplasmic shuttling of Ste11 is regulated by both nutrient- and pheromone-dependent phosphorylation of Ste11^23^. Ste11 is phosphorylated by Pat1, which inhibits Ste11 nuclear localization and transcriptional activity in Rad24, a 14-3-3 protein, dependent manner^8,11,23^. Both PKA and Tor2 also coordinate to inhibit the nuclear accumulation of Ste11 but the molecular mechanism is unclear^24–26^.

Further, Cdk1 phosphorylation of Ste11 inhibits its DNA binding activity and Ste11 is periodically synthesized during the cell cycle. This ensures that only the starved cells arrested in G1 initiate the differentiation program^27^. On the other hand, Ste11 is phosphorylated by Spk1, which is part of Byr2-Byr1-Spk1 pathway activated by the binding of pheromone to its receptor^28^. This promotes the nuclear accumulation of Ste11 and activation of pheromone-induced transcriptional program. The co-expression of h+ specific, *mat1-Pm* and h− specific, *mat1-Mm* in zygotes induces the expression of the Pat1 inhibitor *mei3* ^11^. The expression of constitutively activated Byr2 (MAP3K) induces haploid meiosis^28^. Further, the Sty1 MAPK pathway also promotes mitotic onset in nutrient poor conditions^29^. Recently, Tor2 inactivation is shown to activate Greatwall (Ppk18) mediated phosphorylation of Endosulfine (Igo1) and inhibition of PP2A:B55 to advance mitosis^30^. Both nitrogen poor and starvation conditions are shown to act via Ppk18-Igo1 pathway. These studies reveal that the response of the fission yeast to nutritional shifts is complex and involve intricate regulatory network, which is difficult to understand by intuition alone. Therefore, we adopted a mathematical modelling approach to study meiotic entry in the fission yeast.

Mathematical models of the core cell cycle network have helped to understand the design principles governing the cell cycle transitions in different organisms^31^. A major challenge is to understand how the cell cycle network is modified by the meiosis-specific regulation to promote meiotic entry and commitment. An earlier model proposed for the fission yeast meiosis did not provide a comprehensive account of various events and compared the simulations with the available experimental observations (Table 1)^32^. Therefore, a mathematical model of the fission yeast meiotic entry and commitment network was developed that integrates the current mechanistic knowledge. The model captures the phenotypes of various experimental data (single and multiple mutations) and describes the dynamics of ordering conjugation and meiotic entry. Our analysis revealed that the feedback loop regulation between Ste11 and Pat1 mediated via pheromone signalling (PheS) makes the activation of Mei2 bistable. Further, we show by integrating the meiosis-specific regulation with a cell cycle model, the dynamics of cell cycle exit and entry into meiosis under nitrogen starvation.

## Results

### Modelling Ste11-Mei2-Pat1 subsystem

**Figure 2:**
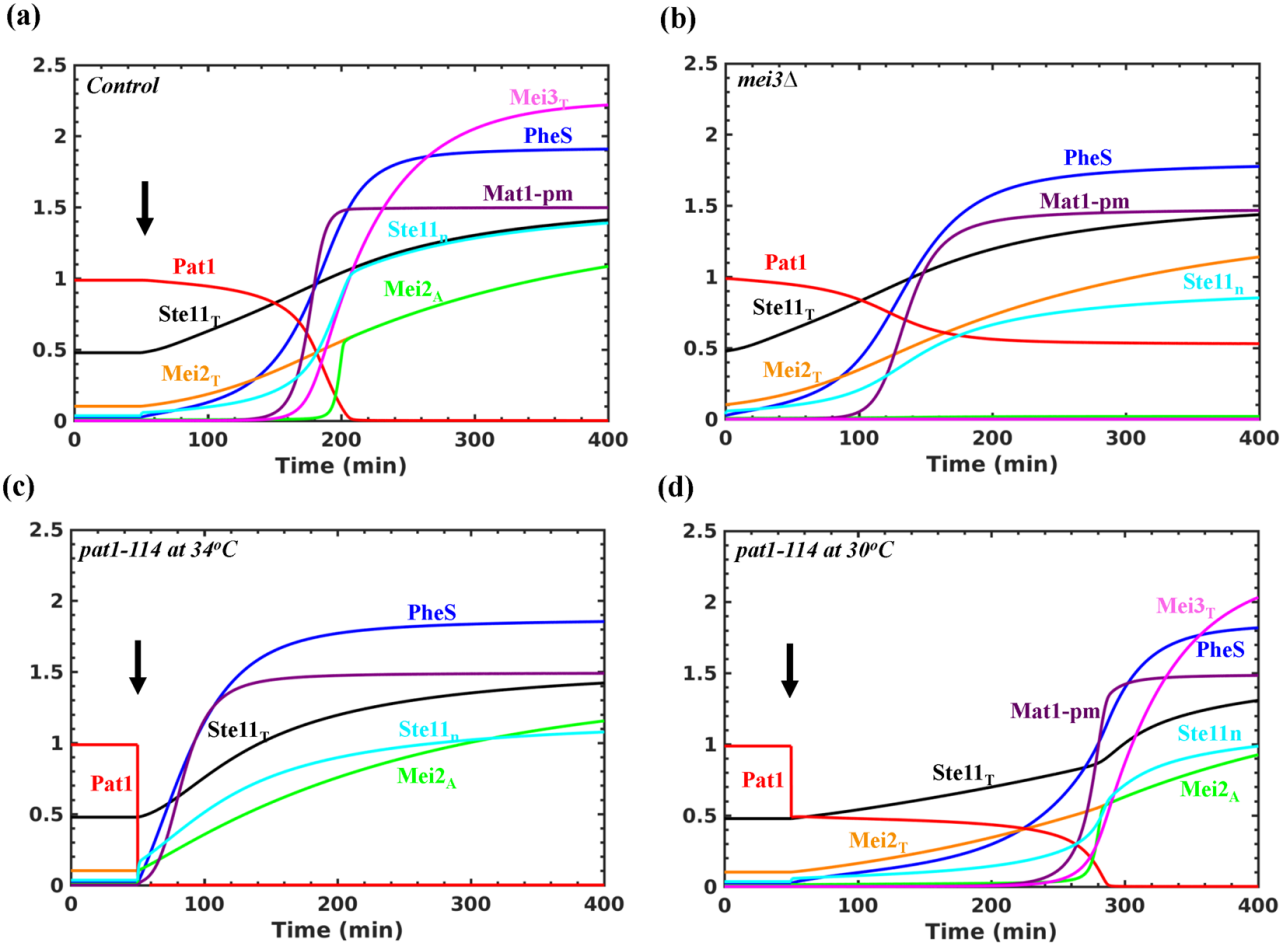
Dynamics of Ste11-Pat1-Mei2 subsystem. (**a**) Control, (**b**) *mei3∆* (k_smei3_ = 0), (**c**) *pat1-114 at 34* °*C* (Pat1_T_ = 0.001, k_smei3_ = 0) and (**d**) *pat1-114 at 30* °*C* (Pat1_T_ = 0.5). (**a**) and (**b**) represent nitrogen starvation (Tor2 = 0, PKA = 0.75), and (**c**) and (**d**) represent nutrient rich conditions (Tor2 = 1, PKA = 1). The arrow indicates the time when both Tor2 and PKA are inactivated.

At first, a mathematical model of the subsystem involving Pat1 inactivation and synthesis/activation of Ste11 and Mei2 was developed (Fig. 1a). Tor2 and PKA were treated as inputs to the module. Their activities represent the nutritional status and it was taken to be high or low depending on whether the cells are grown under nutrient rich or starvation conditions, respectively. *ste11* transcription was considered to be constitutively activated in the absence of Cdk1 regulation of Ste11. This is based on the evidence that *ste11* ^*T82A*^ cells show higher Ste11 levels but they still require nitrogen starvation to undergo differentiation^27^. Figure 2a shows initially the steady state of the subsystem for high Tor2 and PKA activities. Here, the Ste11 levels are slightly higher and Pat1 is active. After 50 min, we decreased the Tor2 and PKA activities, which results in the synthesis of Ste11 and its targets, and the downregulation of Pat1. The upregulation of PheS, Mat1-Pm, Mei3 and active Mei2 occurs sequentially. A higher Ste11 activation threshold for Mat1-Pm ensures that it is synthesized only with the increase in Ste11 by PheS. The time required to activate Mei2 is sensitive to Ste11 and PheS parameters values, and it is known to vary in experiments (2 to 6 hrs) since the wild type (h90) meiosis is asynchronous under nitrogen starvation^5,23,24^. We show that either decrease in PKA or Tor2 activity is sufficient to increase the Ste11 levels and activate Mei2 in the PheS-dependent manner (Figure S1a and b). This is consistent with observations that a mutant defective in either PKA (*pka1∆*/*cyr1∆*) or Tor2 (temperature sensitive *tor2-51*) initiates differentiation under nutrient rich conditions^24,33^. In *pka1∆*, Mei2 activation is rapid in comparison to *tor2-51* as observed experimentally (Figure S1b). Further, Ste11 levels are higher in *pka1∆* compared to *tor2-51*.

In Fig. 2a, the initial rise in Ste11 depends on its synthesis by Rst2 and Ste11 nuclear (Ste11n) accumulation. The Tor2 and PKA inactivation partially relieve the Pat1-dependent inhibitory effects, which upregulate PheS. In turn, PheS promotes further Ste11n accumulation by increasing the nuclear import rate and by inhibiting Pat1 (Fig. 1a). We observe that these feedback loops act redundantly to control Ste11 localization (Figure S2). This is consistent with the observation that the mutation of Spk1 phosphorylation sites in Ste11 does not impair meiotic entry (*ste11* ^*T305A*,*T317A*^)^28^. However, the nuclear import of Ste11 by PheS controls the timing of Mei2 activation (Figure S2a). Further, we observe that Mei2 levels increase initially due to the change in its stability with the Tor2 inactivation and increase further with the Ste11n accumulation. In the absence of Mei2, PheS is not upregulated and the Ste11n accumulation is affected (Figure S3a). This is due to the absence of Mei2-dependent positive feedback loop on the synthesis of Ste11 via the activation of RNA polymerase II. Further, in the absence of either Mei2 phosphorylation by Pat1 (*mei2-SATA*) or Tor2 (*mei2-8A*), Ste11 and Mei2 are expressed/activated in the PheS independent or dependent manner, respectively (Figure S3b and c). Consistently, decreasing the degradation rate of Mei2 (*mts2∆*) leads to the upregulation of PheS and entry into meiosis under nutrient rich conditions (Figure S3d)^19^.

Although PheS inhibits Pat1 activity, this is insufficient to dephosphorylate and activate Mei2 towards meiosis. We observe that the PheS-dependent upregulation of Mei3 (conjugation) is required to inhibit Pat1 completely. In the absence of Mei3, Pat1 kinase activity is at an intermediate level, which prevents the dephosphorylation of Mei2 and entry into meiosis (Fig. 2b). However, the synthesis of Ste11 or PheS upregulation is unaffected. This is consistent with the observation that *mei3∆* cells undergo conjugation but fails to enter meiosis. This suggests that altering the Pat1 inactivation dynamics can alter the order of conjugation and meiosis. We show that with the complete inactivation of Pat1, Mei2 is activated independent of nutritional status and Mei3 (Fig. 2c). On the other hand, the partial inactivation of Pat1 leads to the activation of Mei2 in the Mei3-dependent manner (Fig. 2d). Further, if the extent of Pat1 inhibition by PheS increases, then Mei2 is activated independent of Mei3 (Figure S2d). These observations are consistent with experimental findings using temperature sensitive *pat1-114* (30 °C and 34 °C) and constitutively activated version of the pheromone-responsive Byr2 (*byr2-∆N*)^28,34^. In the latter case, PheS might become hyperactive and inactivate Pat1 to a level that promotes Mei2 activation. Therefore, we conclude that the step-wise inactivation of Pat1 is required for ordering conjugation and meiosis. By coupling the conjugation product, Mei3 to the complete inhibition of Pat1 and activation of Mei2, a strict order of events is achieved.

Further, we studied the role of other kinases, Tor2 and PKA, in suppressing conjugation and meiosis. The activated Tor2 mutant (*tor2-s65*) is sterile and suppresses the effects of nitrogen starvation. We explain the observed behaviour by assuming that the PKA activity is not completely inhibited under nitrogen starvation (see Table S1). However, expression of Mei2 results in the upregulation of PheS and inactivation of Pat1 as shown in the experiments (Figure S4a)^19^. Interestingly, the Tor2 overexpression is also shown to suppress the *pat1-114* meiosis by interfering with Ste11 and Mei2 functions^24^. However, the exact mechanism is unknown. Therefore, we explain the observed behaviour by assuming that the Tor2 overexpression increases both the nuclear export of Ste11 and the degradation of Mei2 to inhibit *pat1-114* meiosis (Figure S4b). Further, we analysed the effect of increasing/decreasing PKA activity (resembling constitutive activation, *cgs1∆* or inactivation, *pka1∆* experiments) with respect to different Tor2 activities (resembling the inactivation, *tor2-51* or overexpression, *nmt-tor2* experiments)^26^. We show that increasing the PKA activity blocks the accumulation of Ste11 with the Tor2 inactivation (Figure S4c). On the other hand, decreasing the PKA activity overcomes the inhibition on Ste11 by Tor2 overexpression, which suggests that both Tor2 and PKA act cooperatively to control Ste11 (Figure S4d). We observe that constitutively activating/inactivating PKA results in very low/high basal Ste11 levels, respectively. This can either suppress or promote differentiation irrespective of the Tor2 activity.

We tested the sensitivity of the model to the nuclear import and export rate of Ste11. We observe that either blocking the export or increasing the import leads to an increase in Ste11 and meiotic entry in nutrient rich conditions (Figure S5). The experiments have shown that blocking the export of Ste11 from the nucleus by addition of leptomycin B (LMB) leads to Ste11n accumulation^23^. Further, cells carrying mutation that mimic Ste11 phosphorylation by Spk1 (*ste11* ^*T305D*,*T317D*^) enter meiosis in the presence of nitrogen^28^. Also, the Ste11 overexpression induces Mei2 activation in nutrient rich conditions (data not shown).

### Bifurcation analysis of Mei2-Ste11-Pat1 subsystem

**Figure 3:**
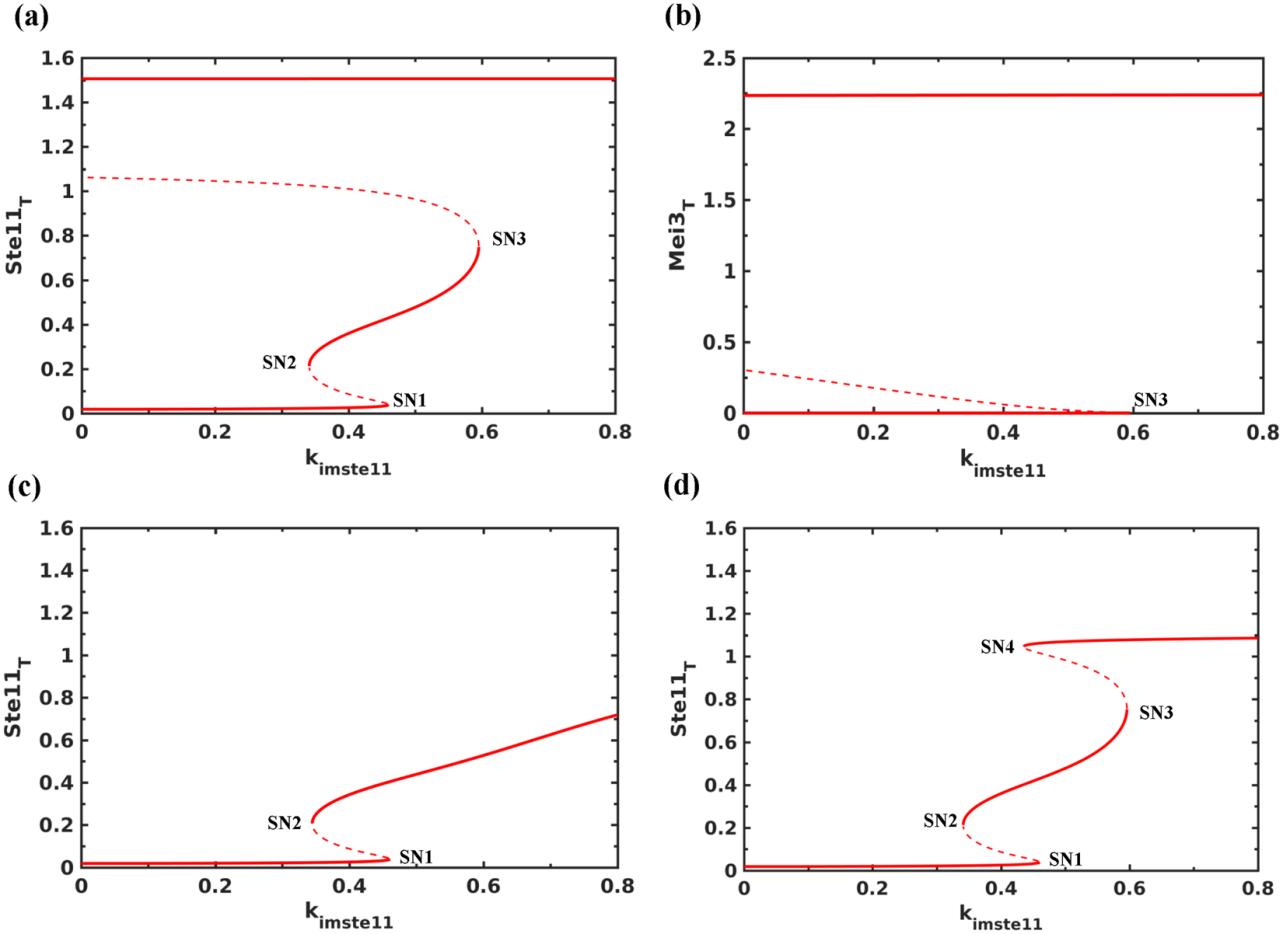
Bifurcation analysis of Ste11-Mei2-Pat1 subsystem. The effect of increasing the nuclear import rate of Ste11 (k_imste11_) under nitrogen rich conditions (Tor2 = 1, PKA = 1) on (**a**) Ste11_T_, (**b**) Mei3_T_, (**c**) in the absence of PheS (k_sphe_ = 0) and (**d**) *mei3∆* (k_smei3_ = 0). SNs represent Saddle Nodes, solid lines represent stable steady states and dashed lines represent unstable steady states.

We carried out the bifurcation analysis to understand the effect of different feedback loops and sensitivity of the model to Ste11 parameter values (see equations in supplementary information). We computed the effect of changing the nuclear import rate of Ste11 (*k*_*imste11*_ in the model) on the activation/expression of Ste11 and Mei2 since the ratio of import to export rate changes under nitrogen starvation. Figure 3a shows that with the increase in nuclear import rate of Ste11 there is a sequential activation of two bistable switches: one is reversible and other is irreversible. These switches establish Ste11 low, intermediate and high steady states and depend on multiple feedback loops acting on the Ste11 nuclear import/export and synthesis. We found that the autoregulation of Ste11 synthesis establishes the intermediate state (between SN1 and SN2) at lower import rate rates and it is sensitive to the half-saturation constant (*k*_*mste11*_) and Hill coefficient (*hs*) in the rate expression.

**Figure 4:**
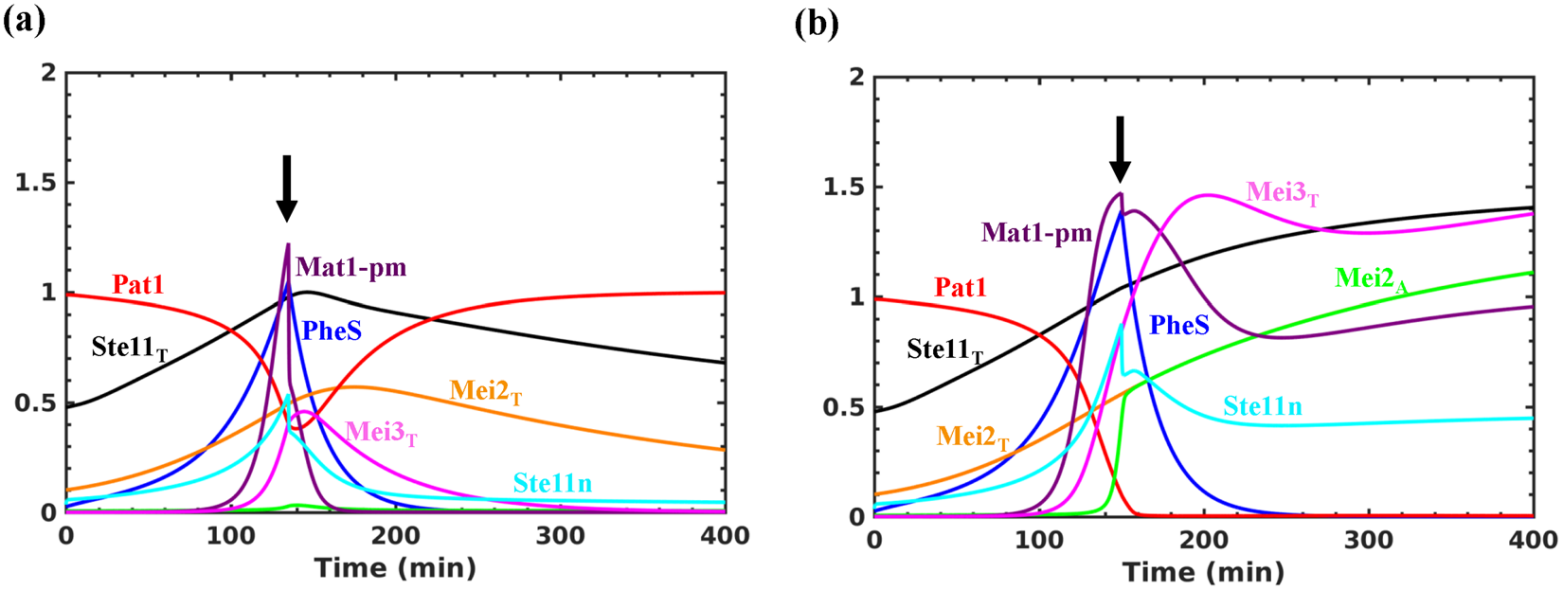
Testing the irreversibility of meiotic entry. Tor2 and PKA are inactivated initially and after Mat1-Pm synthesis they are re-activated at either (**a**) early or (**b**) late time point. The arrow indicates the time when Tor2 and PKA are re-activated (Tor2 = 1, PKA = 1) and PheS (k_sphe_ = 0) is inactivated.

At a higher import rate (SN3), we found that the upregulation of PheS contributes towards an increase in Ste11n accumulation. This leads to an increase in the accumulation of Mei2 and Mei3 (Fig. 3b). We observe that the increase in Mei2 is also accompanied by the accumulation of dephosphorylated Mei2. The irreversible activation of Mei2 signals the point of no return to mitosis and serves as a commitment point. In the absence of PheS, the expression and activation of Mei2 switch is affected (Fig. 3c). *mei3∆* also blocks Mei2 activation but does not inhibit Ste11 and Mei2 accumulation, and exhibits a bistable characteristic (Fig. 3d). This depends on the feedback loops acting between Ste11 and PheS: Ste11n upregulates PheS and PheS promotes Ste11n accumulation. However, in the absence of Mei3, the switch becomes reversible. This suggests that PheS dependent accumulation of Mat1-Pm and Mei3, and inhibition of Pat1 contribute to make the transition to meiosis irreversible. We observe that PheS is only required to promote the transition but is not required to maintain it. Inactivating PheS after the transition does not lead to the re-activation of Pat1 (data not shown). Further, activating Tor2/PKA and inactivating PheS, mimicking the return to growth experiments, at early (135 min) and late (150 min) time points after the increase in Mat1-pm levels result in the reversible and irreversible inactivation of Pat1, respectively (Fig. 4a and b). This represents the window of opportunity for the cells to return to mitosis after conjugation (diploid mitosis) and it is sensitive to the synthesis rate of Mei3. Such a short time window reduces the chance of return to growth as diploids. Further, the intermediate state of Ste11 at lower import rates corresponds to the state of the cell cycle since meiosis regulators are not activated and might become unstable during the cell cycle to exhibit Ste11 oscillations as observed experimentally (see the next section).

### Integrated model of mitosis to meiosis transition

**Figure 5:**
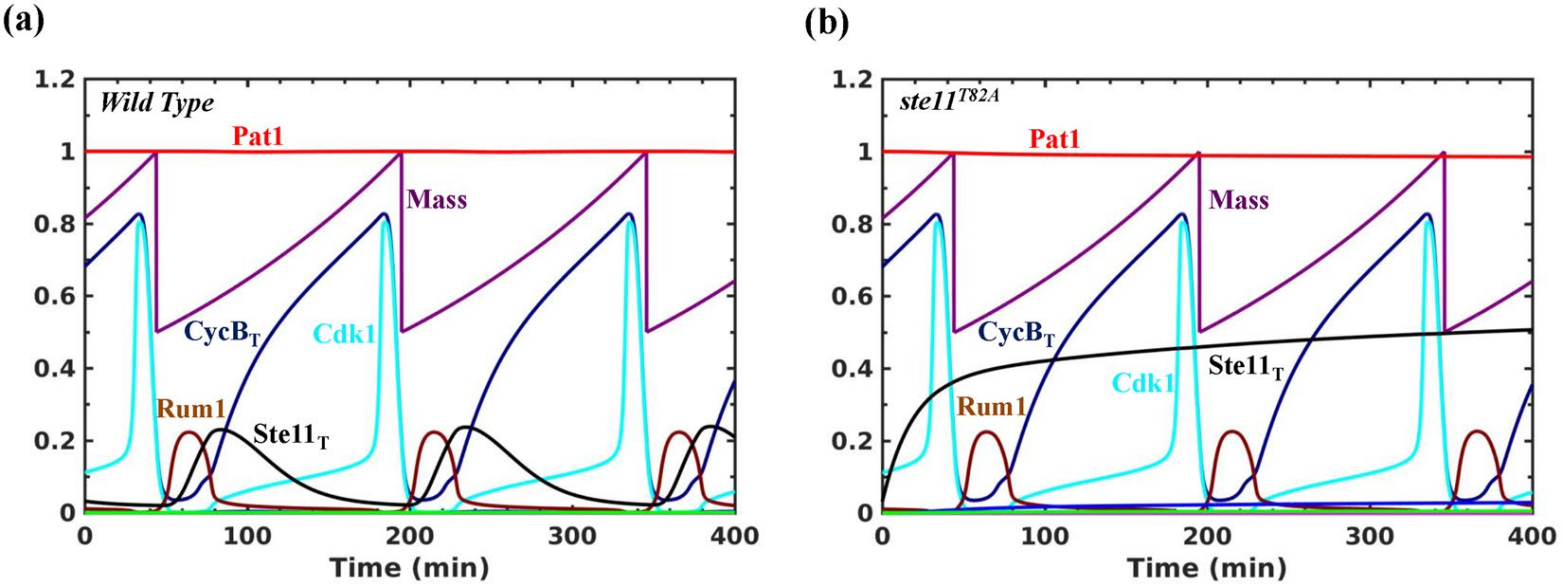
Dynamics of Ste11 during the fission yeast cell cycle. (**a**) Wild type (**b**) *ste11* ^*T82A*^.

We integrated the meiosis model with an existing model of the fission yeast cell cycle (Fig. 1b)^30^. Here, Cdk1 inhibits Ste11 binding to its promoter. A dynamic simulation of the fission yeast cell cycle shows that the Ste11 accumulation is periodic depending on the Cdk1 oscillation (Fig. 5a). Ste11 is high when Cdk1 is low and activates its own synthesis. The inhibition of Ste11 synthesis by Cdk1 leads to its disappearance since it is a highly unstable protein. In the absence of Cdk1 regulation of Ste11, its levels increase but does not lead to the activation of Mei2 as shown in the experiments (*ste11* ^*T82A*^) (Fig. 5b).

**Figure 6:**
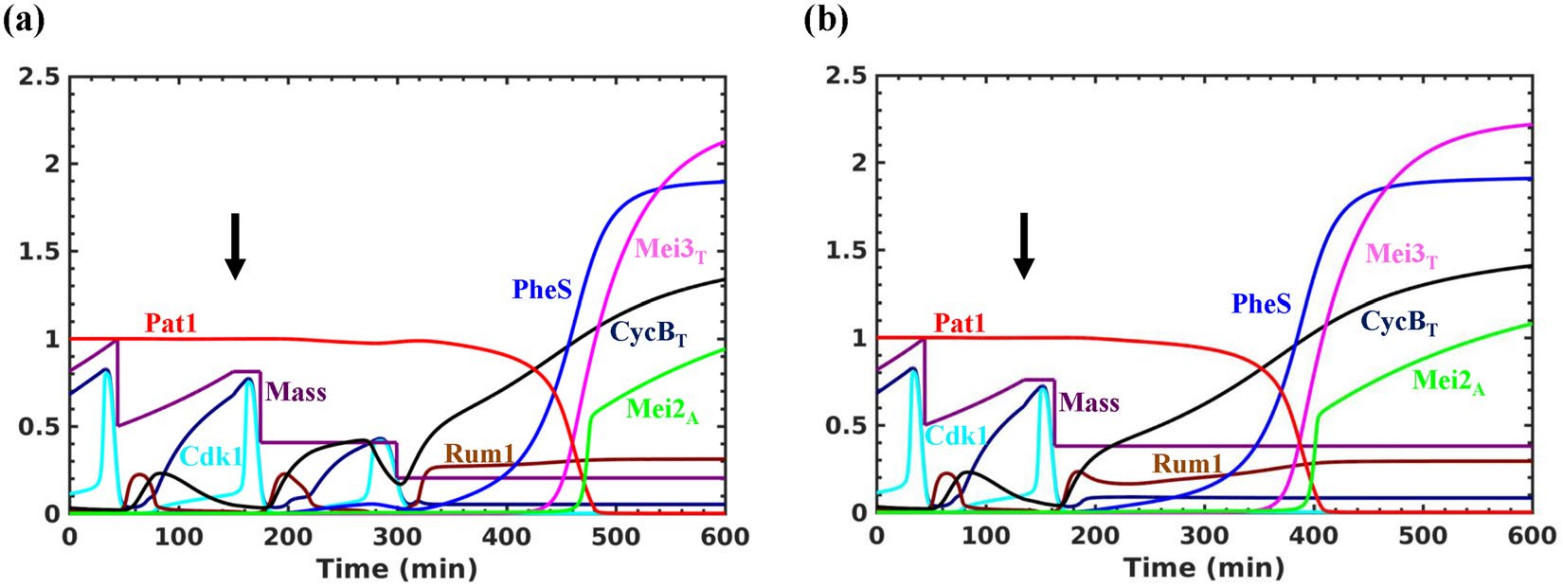
Dynamics of mitosis to meiosis transition under nitrogen starvation. Both Tor2 and PKA are inactivated at two different time points of the cell cycle: (**a**) 150 min and (**b**) 135 min (Tor2 = 0, PKA = 0.75). The arrow indicates the time when both Tor2 and PKA are inactivated.

We explored how cells undergo two accelerated cell divisions and arrest in G1 under nitrogen starvation. It is still not clear how cells divide when there is growth inhibition. It is postulated to occur through recycling of intracellular nitrogen sources^35^. Therefore, we included its contribution for the synthesis of cyclins under nitrogen starvation. Further, both nitrogen starvation and pheromone signalling independently are shown to induce G1 arrest/delay^36–38^. Interestingly, studies have shown that Ste11 is required for the pheromone induced G1 arrest and the addition of pheromone to *cyr1∆* cells accelerate M-phase^28,36,38^. On the other hand, the transcription of pheromone dependent genes takes place in G1, which restricts the meiotic program to start from the G1 window of the cell cycle. Therefore, we also explored the role of PheS in G1 arrest. Figure 6a shows that the Tor2 and PKA inactivation leads to two accelerated cell divisions (in the absence of growth) followed by G1 arrest with the high Rum1 activity. There is an early increase in Ste11 followed by the upregulation of PheS. A complete inactivation of Pat1 in G1 leads to the activation of Mei2 and commitment to meiosis. In enlarged G2 cells, there are two step-wise reductions in the cell size which is accompanied by the growth inhibition (Fig. 6a). In smaller G2 cells, nitrogen starvation leads to an accelerated entry into M-phase and arrest in G1 after the first division (Fig. 6b). In this case, the G1 arrest is dependent on PheS, which is known to have an effect on the cell cycle only in smaller cells^36^. In the absence of PheS, we observe another round of cell division (data not shown).

### Bifurcation analysis of the integrated model

We performed the bifurcation analysis of the integrated model to understand the size-control mechanisms under nitrogen starvation. The size control in the fission yeast operates at the G1/S and G2/M boundary. The fission yeast growing on nitrogen rich conditions produce daughter cells with a size greater than the threshold required to initiate S phase, which makes the G1/S size control cryptic. However, the G1/S control becomes pertinent in the cells grown under different conditions: nitrogen poor medium, nitrogen starvation or in the presence of pheromone.

**Figure 7:**
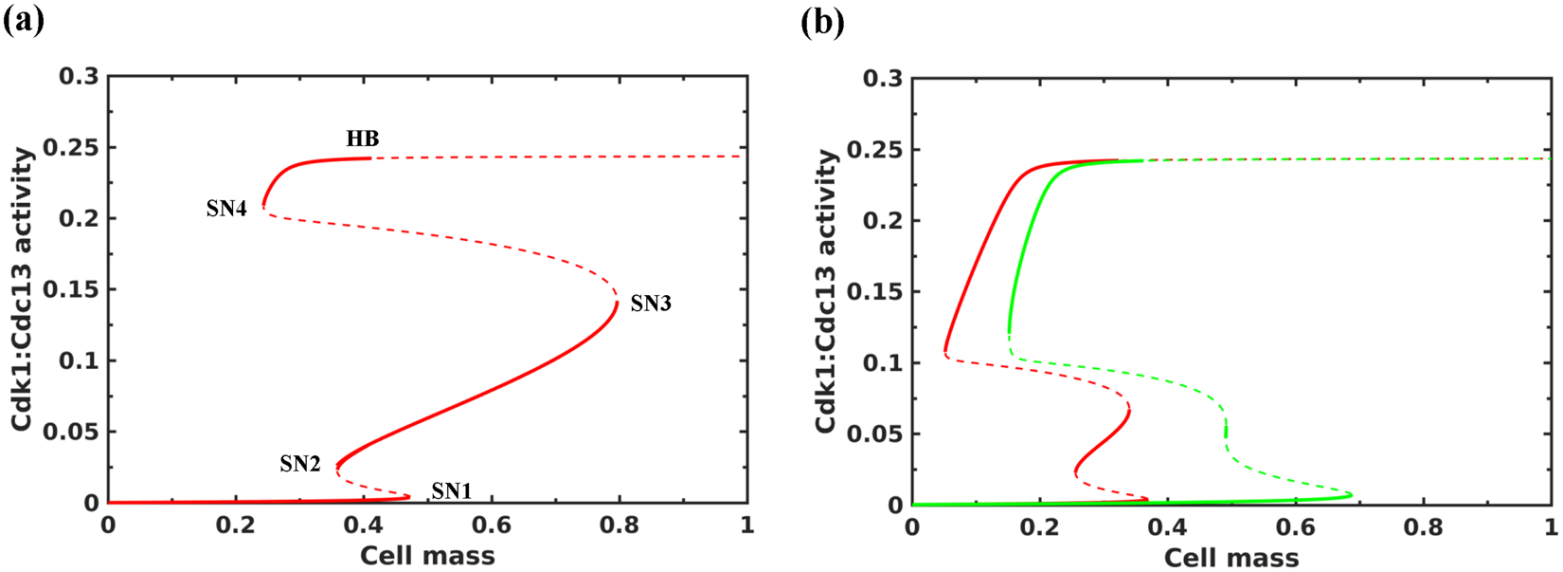
Bifurcation analysis of the integrated model. Cdk1:Cdc13 activity is plotted as a function of the cell mass for (**a**) cells under nitrogen rich conditions, and (**b**) cells under nitrogen starvation in the absence (red line) and presence (green line) of PheS. HB represents Hopf Bifurcation, SNs represent Saddle Nodes, solid lines represent the stable steady states and dashed lines represent the unstable steady states.

A plot of Cdk1 activity as a function of the cell size yielded three stable steady states (solid lines) corresponding to the phases of the cell cycle as shown by Chica *et al*.^30^ (Fig. 7a). The low Cdk1 phase at small cell sizes corresponds to G1 phase established by the antagonism between Rum1 and Cdk1. The intermediate Cdk1 phase corresponds to S/G2 established by the inhibitory phosphorylation of Cdk1. The high Cdk1 phase corresponds to M phase, which becomes destabilized by the activation of ubiquitin ligase, Anaphase Promoting Complex/Cyclosome (APC/C), dependent degradation of Cdc13 (Cyclin B), an activating partner of Cdk1. There are two size thresholds at low and intermediate Cdk1 activities corresponding to S and M phase entry, respectively. Figure 7b shows that the intermediate steady state corresponding to the S/G2 phase disappears with the Tor2 and PKA inactivation, which promotes the mitotic onset at the smaller cell size. In the absence of PheS, the G1/S size threshold decreases in comparison to nutrient rich conditions (red line in Fig. 7b). This is due to the intracellular nitrogen sources contributing for the synthesis of cyclins. On the other hand, in the presence of PheS, the G1/S size threshold increases in comparison to nutrient rich conditions (green line in Fig. 7b). Cells undergoing two cell divisions escape the G1/S checkpoint after the first division since their cell sizes are well above the G1/S size threshold. On the other hand, smaller cells when they divide, their cell sizes are closer to G1/S size threshold. This slows down their entry into mitosis and provides a window of opportunity for PheS to establish G1 arrest by increasing the G1/S size threshold.

## Discussion

In this study, the dynamics of mitosis to meiosis transition in the fission yeast was explored. We assembled the interaction network of meiotic entry based on the literature spanning few decades. Mathematical modelling of this network revealed how these feedback loops are coupled. We showed that the mitosis to meiosis transition in the fission yeast is governed by a bistable switch, which ensures the irreversible commitment to meiosis (Fig. 3). The double negative feedback loop between Ste11 and Pat1 mediated via conjugation product Mei3 (Ste11n → Mat1-Pm → Mei3 ┤ Pat1 ┤ Ste11n) makes the transition irreversible. The transition requires nitrogen starvation (Tor2/PKA inactivation)- and PheS-dependent increase in the nuclear import and synthesis of Ste11, which promote the synthesis of Mei3 and the complete inactivation of Pat1.

We demonstrated that the meiotic switch is sensitive to different parametric alterations. There is a coordination of different kinases, Pat1, Tor2, PKA and Cdk1 acting on the same targets (crosstalk) to suppress the switch (Figs 2 and S1). Recent evidences show that Pat1 and Tor2 act on the same targets, Ste11 and Mei2^8,19^. This suggests that there is a combinatorial control to keep the switch inactive. On the other hand, the activation of Ste11 also requires the coordination of kinases, Sty1 and Spk1. The synergistic action is encoded by the control of transcriptional activators (activation and localization), and by the control of RNA polymerase II to drive the synthesis of Ste11.

We showed that feedback loops acting on Ste11 are triggered by either increasing the nuclear accumulation or the Ste11 concentration (Figure S5). Both these effects are promoted by the Tor2 and PKA inactivation. In the model, the PKA inactivation increases the synthesis of Ste11 via Rst2 while the Tor2 inactivation increases it via Mei2. Both Tor2 and PKA also control the Ste11 localization. Thus, Tor2 and PKA forms coherent feedforward loops (CFFLs) that provide a robust restraint on meiosis controls in mitosis (Fig. 1a). Tor2/ PKA-dependent CFFLs are coupled to positive feedback loops acting on Ste11. These factors helped to simulate the experimental data showing the entry into meiosis with inactivation of either Tor2 or PKA, and with *mei2-8A* (Figures S1 and S3). In *mei2-8A*, an inhibitory branch of CFFL is compromised leading to delayed activation of Mei2. The inactivation of both these kinases acts as ‘trigger’, while the inactivation of Pat1 is required to enter and establish the meiotic state. The feedback loops acting on the Ste11 localization and synthesis are coupled to the activity of Pat1 and hence overcoming or inhibiting its activity becomes crucial. Therefore, the Tor2 and PKA inactivation is dependent on PheS to overcome the Pat1 inhibition. The incomplete inactivation of Pat1 bypasses the requirement of PKA and Tor2 inactivation but it also depends on PheS to inhibit the remaining Pat1 activity (Fig. 2d). This suggests that the PKA and Tor2 inactivation promote a step-wise reduction in the Pat1 activity.

Our analysis showed that PheS is required for meiotic entry but is not required to maintain the state (Figs 3c and 4b). This might be relevant considering that the PheS activation can be transient due to the negative feedback loop regulation^39^. We also showed that increasing the PheS activity can make the transition Mei3 independent by inhibiting the Pat1 activity completely (Figure S2d). This suggests that controlling PheS pathway activation is also crucial for ordering conjugation and meiosis. The PheS-dependent feedback loops, Ste11n → PheS → Ste11n and Ste11n → PheS ┤ Pat1 ┤ Ste11n are capable of making the system bistable, but their activation is insufficient to activate Mei2 (Fig. 3d). The activation of these feedback loops promotes the activation of the double negative feedback loop: Ste11n → Mat1-pm → Mei3 ┤ Pat1 ┤  Ste11n, for the irreversible activation of Mei2 (Fig. 3b). This double negative feedback loop forms a CFFL with Mei2 and influences its activation dynamics (Fig. 1a). The complete inactivation of Pat1 leads to premature activation of Mei2 (Fig. 2c). The sustained nuclear accumulation of Ste11 ensures that the synthesis of Ste11, Mei2 and Mei3 are maintained for the meiotic commitment with re-addition of nitrogen (return to growth experiment) (Fig. 4a).

In nutrient rich conditions, we showed that the Ste11 accumulation is periodic due to the regulation of Ste11 synthesis by Cdk1 (Fig. 5). On the other hand, the Tor2 and PKA inactivation accelerated the entry into M phase with step-wise reduction in the cell size by two cell divisions (Fig. 6). This established the cell cycle arrest in G1. The bifurcation analysis showed that the G2/M size threshold is decreased and the G1/S size threshold is influenced by the increase in intracellular nitrogen sources under nitrogen starvation and PheS (Fig. 7). A decrease in the G1/S size threshold helped bigger cells to divide the second time and reduce their cell size further in preparation for quiescence in the absence of mating partners. In these cells, we observed that PheS is unable to establish the cell cycle arrest after the first division. However, in smaller G2 cells, PheS established G1 arrest due to the delay in entering the second division. It is known that addition of pheromones to cells in G2 accelerate mitosis and induce G1 arrest after the first or second division depending on the initial cell size^36^. A recent study also claimed that nitrogen starvation can transiently block the mitotic onset followed by two fast rounds of cell division. In this scenario, the transient block can extend the G2/M size threshold and might provide an opportunity for cells to increase their size and undergo two cell divisions^40,41^. Further experiments are required to establish the role of intracellular nitrogen sources and transient block of mitotic onset in the step-wise reduction of the cell size under nitrogen starvation.

The cell cycle arrest in G1 is followed by commitment to meiosis with the inactivation of Pat1 and the activation of Mei2. It is shown that the fission yeast commits to meiosis at the G1/S boundary in return to growth experiments^1,5^. On the other hand, in the budding yeast, the commitment to meiosis occurs at the G2/M boundary^42^. We previously developed a mathematical model of meiosis commitment point in the budding yeast^43^. We showed that the double negative feedback loop between Ama1 (APC/C activator) and Cdk1 contributes towards the irreversible entry into meiosis. This is also accompanied by the autoregulation of Ndt80, a meiosis-specific transcriptional activator in the budding yeast. Ndt80 is also regulated by transcription repressor Sum1, which is in turn regulated by Ndt80 targets forming multiple feedback loops in the network. Here, Ndt80 is sensitive to both the recombination checkpoint and nutritional input (via Ime2). Therefore, in both yeasts, we find that inhibitor and activator of the transition are locked in feedback loops that ensure an irreversible entry under nitrogen starvation (fission yeast) or recombination checkpoint (budding yeast) inactivation. This is similar to the regulatory design proposed for mitotic transitions recently^44^.

In mammals, meiotic initiation is controlled by retinoic acid (RA) induced expression of *STRA8* ^45^. The RNA binding protein Dazl is required to enable germ cells to respond to RA. Further, meiotic progression depends on the RNA binding protein Meioc^46^. The functions of these RNA binding proteins resemble the Mei2 function in the fission yeast. However, the regulatory mechanism inter-linking these molecular players is largely unknown. On the other hand, in *Caenorhabditis elegans*, it is shown that the mitosis to meiosis transition is controlled by double negative feedback loops between RNA binding GLD proteins and regulators that promote germline self-renewal^47^. This suggests that the mutual antagonism between regulators of the cell cycle and meiosis might be a conserved feature driving the irreversible meiotic transition.

### Limitation and future work

In this work, we made a first attempt to model the Ste11 regulation to capture the qualitative behaviour of various mutants. The next step is to test various models of multi-site phosphorylation of Ste11. Further, Ste11 is also transcriptional regulated by Atf1, Pcr1 and Gaf1. Atf1 and Pcr1 are known to act via stress MAPK, Sty1 and Gaf1 is a transcriptional repressor. The transcription of *ste11* is also regulated by SAGA (Spt–Ada–Gcn5–acetyltransferase), a co-activator recruited by Rst2. The molecular basis of these regulations are still unclear^15^. Recently, SAGA associated Ubp8 is shown to be involved in the histone H2B deubiquitylation, which favours RSC dependent chromatin remodelling and synthesis of Ste11 in Pol II CTD phosphorylation dependent manner^48^. It will be interesting to further decipher by modelling and experiments the combinatorial control of Ste11 in driving the switch from proliferation to differentiation.

## Methods

The fission yeast regulatory network of meiotic entry and commitment is shown in Fig. 1a. The network was translated into a set of non-linear ordinary differential equations (ODEs), which describes the dynamics of individual components (see Supplementary information). Here, we modelled only the transcriptional and post transcriptional regulation of Ste11 controlled by nutritional inputs acting via Tor2 and PKA. The Ste11 module includes the regulation of Ste11 synthesis by Rst2, Ste11n, and RNA polymerase II (Rpol), and the regulation of Ste11 localization by Pat1, PKA and Tor2 in a cooperative manner. The Mei2 module includes the regulation of Mei2 synthesis and degradation, Mei2 multi-site phosphorylation by Pat1 and Tor2, and Rpol regulation by Mei2. The Pat1 module includes the regulation of Pat1 by PheS and stoichiometric inhibitory complex formation between Pat1 and Mei3. Further, the model includes the upregulation of PheS and synthesis of Mat1-Pm by Ste11n, and the synthesis of Mei3 by Mat1-Pm (in the presence of opposite mating cell type).

Multi-site phosphorylation and dephosphorylation of Mei2 are described using Michaelis-Menten kinetics and Ste11-dependent synthesis/upregulation of components (PheS, Mei2, Ste11 and Mat1-Pm) are described by Hill equations, while all other reactions are represented by the law of mass action (see supplementary information). We used Hill equations to eliminate the intermediate steps in the activation of PheS and conjugation. The half-lives of Ste11 and Mei2 (both phosphorylated and unphosphorylated forms) were calculated from the experimental data in the literature^8,19^. Other model parameter values were obtained by simulating the model to capture the qualitative behaviour of various mutants (Table 1 and S1). Such an approach was used previously to model the budding yeast cell cycle and meiosis commitment^43,49,50^.

**Table 1:** List of experimental situations analysed to develop the meiosis model.

Strain	Mating	Meiosis	Strain	Mating	Meiosis
**Homothallic** (**h90**)			**Homothallic** (**h90**)		
**Nitrogen rich**			**Nitrogen starved**		
*pka1∆/cyr1∆*	+	+	Wild type(WT)	+	+
*tor2-51*	+	+	*rst2∆*	−	−
*tor2-51cgs1∆*	−	−	*cgs1∆*	−	−
*tor2-51pka1∆*	+	+	*mei2∆*	−	−
*ste11 overexpression*	+	+	*mei3∆*	+	−
*ste11* ^*T82A*^	−	−	*mat1pm∆*	−	−
*ste11* ^*T173A*,*S218A*^	−	−	*spk1∆*	−	−
*ste11* ^*T305D*,*T317D*^	+	+	*ste11∆*	−	−
*WT in leptomycin B*	+	+	*ste11* ^*T173D*, *S218D*^	−	−
*pat1-114 at 30 * ^*o*^ *C*	+	+	*ste11* ^*T305A*, *T317A*^	+	+
*mts2∆*	+	+	*ste11* ^*T82D*^	−	−
*tor2-ts6 mei2∆*	−	−	lsk1∆*	−	−
*byr2-∆N*	−	+	*nmt1-ste11 lsk1∆*	+	+
*byr2-∆N mei3∆*	−	+	*nmt-tor2*	−	−
*mei2-8A*	+	+	*nmt-tor2 cyr1∆*	+	+
*mei2-8A-SATA*	−	+	*tor2-s65*	−	−
			*tor2-s65 pREP41-mei2*	+	+
**Heterothallic** (**h+ or h−**)			**Heterothallic** (**h+ or h−**)		
**Nitrogen rich**			**Other conditions**		
*pat1-114* (*34 * ^*o*^ *C*)	−	+	cyr1∆ + M factor		
*mei2∆ pat1-114*(*34 * ^*o*^ *C*)	−	−			
*nmt-tor2 pat1-114* (*34 * ^*o*^ *C*)	−	−			
*ste11∆ pat1-114* (*34 * ^*o*^ *C*)	−	−			
*lsk1∆ pat1-114* (*34* °*C*)	−	−			
*mei2-SATA*	−	+			
*mei2-L-SATA*	−	+			
*mei2-L-SATA lsk1*∆*	−	−			
*pat1*∆mei2*∆nmt1-mei2*	−	+			
*mei2∆nmt1-mei2*	−	−			

− Sign means phenotypic block or low frequency.

Diploid strain (h+/h−) experimental data were also analysed.

*Lsk1 is α subunit of CTDK-I (RNA polymerase II C-terminal domain kinase I).

Further, we integrated the meiosis-specific model with an existing model of the fission yeast cell cycle developed by Novak’s group to understand the dynamics of the cell cycle exit under nitrogen starvation (Fig. 1b)^30^. In addition to the core cell cycle network, this model also includes the regulation of PP2A:B55, a phosphatase that promotes Cdk1 inhibitory phosphorylation, by Ppk18-Igo1 pathway with the Tor2 and PKA inactivation (see supplementary information). The models are integrated through Cdk1-dependent regulation of Ste11 binding to its promoter and PheS-dependent regulation of Rum1 degradation.

The equations and parameter values of the meiosis-specific and integrated models are provided as part of the supplementary information along with the XPPAUT codes. Models were simulated numerically using XPPAUT, available from http://www.math.pitt.edu/∼bard/xpp/xpp.html, to obtain the temporal profiles and bifurcation diagrams.

## Electronic supplementary material

Supplementary Information
