# Supplementary material for: Computational modelling of meiotic entry and commitment
Source: Sci Rep. 2018 Jan 9;8:180. doi: 10.1038/s41598-017-17478-9 (PMC5760542; doi:10.1038/s41598-017-17478-9)
Supplement: Supplementary file 1 — Supplementary Information [file 41598_2017_17478_MOESM1_ESM.pdf]

Supplementary Information

Computational modelling of meiotic entry and commitment

Tanvi Bhola[1], Orsolya Kapuy[2] and P.K. Vinod[1*]

[1] Center for Computational Natural Sciences and Bioinformatics, International Institute of Information Technology, Hyderabad-500032, India

[2]Semmelweis University, Department of Medical Chemistry, Molecular Biology and Pathobiochemistry, Budapest, Hungary

*Correspondence: vinod.pk@iiit.ac.in

Model of meiosis entry and commitment:

Figure 1a shows the interaction network involved in the control of meiotic entry and commitment under nitrogen starvation (Starv). Different signalling cascades are activated/inactivated under nitrogen starvation. Tor2 and cAMP-PKA signalling pathways are inactivated, while stress MAPK and pheromone signalling (PheS) are activated. Here, we focused only on how the dynamics of Ste11-Pat1-Mei2 subsystem is influenced by the activation/inactivation of pathways. The rate of change of concentration with time for each component is described by a nonlinear, ordinary differential equation (ODE). All dynamic variables are dimensionless and represent relative protein concentrations. Rate constants have a dimension of min^{-1}. Michaelis constants, Half-saturation constants and other parameters are dimensionless.

The Ste11 module:

The synthesis of Ste11 is transcriptional regulated by PKA sensitive transcriptional activator Rst2 and by Ste11 itself. We also included the regulation of the Ste11 synthesis by RNA polymerase II (Rpol). The degradation rate of Ste11 was calculated from Kitamura *et al* (2001). The equation for the total concentration of Ste11 (Ste11T) is given below:

$$\frac{dSte11_T}{dt} = \left(k_{sste11} \cdot \frac{k_{rst}}{k_{rst} + PKA} + k'_{sste11} \cdot \frac{Ste11n^{hs}}{k_{mste11}^{hs} + Ste11n^{hs}}\right). \; Rpol - k_{dste11} \, . \, Ste11_T$$

The nuclear localization of Ste11 ($Ste11_n$) is controlled by the ratio of its import to export rates. The Ste11n accumulation is inhibited by Pat1 and nutrient responsive kinases (PKA and Tor2), while promoted by PheS. We considered that Pat1, PKA and Tor2 cooperate to increase the nuclear export rate of Ste11 while PheS increases the nuclear import rate. We also considered that Pat1 controls Ste11n accumulation independently of Tor2 and PKA activity since their inactivation still depends on PheS for the Ste11n accumulation and the complete inhibition of Pat1 bypasses the requirement of PheS [6,7,24]. Pat1 can inhibit Ste11 by inhibitory phosphorylation and by also other unknown mechanisms. This is due to the observation that *ste11*$^{T173A,\, S218A}$ cells still require PheS for the Ste11n accumulation[28].

$$\frac{dSte11_n}{dt} = (k_{imste11} + k'_{imste11} \, . \, PheS). \; (Ste11_T - Ste11_n) - (k_{exste11m} + k_{exste11}.mu + k'_{exste11}. \; Pat1 + k"_{exste11} \, .mu.Pat1) \, .Ste11_n$$

mu is a function of Tor2 and PKA activities. Ste11n accumulation is required for the upregulation of PheS and synthesis of Mat1-Pm and Mei2.

The Mei2 module:

It is shown that both Tor2 (TORC1) and Pat1 cooperate to destabilize Mei2 under nutrient rich conditions [8,9,19]. The mechanism used for modelling Mei2 is given below:

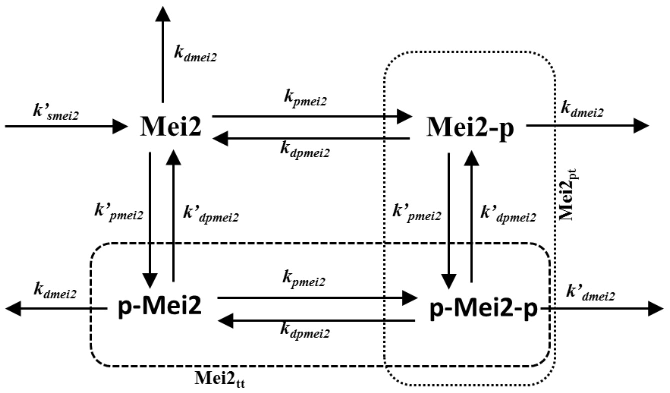

$Mei2_T = Mei2 + Mei2\text{-}p + p\text{-}Mei2 + p\text{-}Mei2\text{-}p$
$Mei2_{pt} = Mei2\text{-}p + p\text{-}Mei2\text{-}p$ (total concentration of Pat1 phosphorylated Mei2 forms)
$Meit2_{tt} = p\text{-}Mei2 + p\text{-}Mei2\text{-}p$ (total concentration of Tor2 phosphorylated Mei2 forms)

The degradation rates of Mei2 was calculated from the Ostubo *et al* (2014) ($k'_{dmei2} > k_{dmei2}$). The synthesis rate (k'_{smei2}) of Mei2 depends on Ste11. Mei2 phosphorylation rates: k_{pmei2} and k'_{pmei2} depend on Pat1 and Tor2 activities, respectively. The multi-site phosphorylation and dephoshorylation of Mei2 are described by Michaelis-Menten kinetics and the equations are given below.

$$\frac{dMei2_{pt}}{dt} = k_{pmei2} \,.\, Pat1 \,.\, \frac{\left(Mei2_T - Mei2_{pt}\right)}{\left(J_{pmei2} + \left(Mei2_T - Mei2_{pt}\right)\right)} - k_{dpmei2} \cdot \frac{Mei2_{pt}}{\left(J_{pmei2} + Mei2_{pt}\right)} - k_{dmei2} \,.\left(Mei2_{pt} - Mei2_{pp}\right) - k'_{dmei2} \,. Mei2_{pp}$$

$$\frac{dMei2_{tt}}{dt} = k'_{pmei2} \,.\, Tor2 \,.\, \frac{\left(Mei2_T - Mei2_{tt}\right)}{\left(J_{tmei2} + \left(Mei2_T - Mei2_{tt}\right)\right)} - k'_{dpmei2} \cdot \frac{Mei2_{tt}}{\left(J_{tmei2} + Mei2_{tt}\right)} - k_{dmei2} \,.\left(Mei2_{tt} - Mei2_{pp}\right) - k'_{dmei2} \,. Mei2_{pp}$$

$$\frac{dMei2_{pp}}{dt} = k_{pmei2} \,.\, Pat1 \,.\, \frac{\left(Mei2_T - Mei2_{pt} - Mei2\right)}{\left(J_{pmei2} + \left(Mei2_T - Mei2_{pt}\right)\right)} - k_{dpmei2} \cdot \frac{Mei2_{pp}}{\left(J_{pmei2} + Mei2_{pt}\right)} + k'_{pmei2} \,.\, Tor2 \,.\, \frac{\left(Mei2_T - Mei2_{tt} - Mei2\right)}{\left(J_{tmei2} + \left(Mei2_T - Mei2_{tt}\right)\right)} - k'_{dpmei2} \cdot \frac{Mei2_{pp}}{\left(J_{tmei2} + Mei2_{tt}\right)} - k'_{dmei2} \,. Mei2_{pp}$$

Pat1, in addition to decreasing the stability of Mei2, also inhibits Mei2 by phosphorylation and blocks the commitment to meiosis. Mei2 is known to act via stress-responsive MAPK

(Sty1) pathway to promote RNA polymerase II (Rpol) CTD phosphorylation[20]. We considered that Mei2 directly controls Rpol activity and both Pat1 and Tor2 are required to inhibit this function of Mei2.

$$\frac{dRpol}{dt} = \left(k_{apol} . + k'_{apol} . (Mei2T - Mei2pp)\right). \ (1 - Rpol) - k_{ipol} . Rpol$$

The Pat1 module:

The Pat1 regulation includes PheS-dependent inactivation of Pat1 and stoichiometric inhibitory complex formation between Pat1 and Mei3.

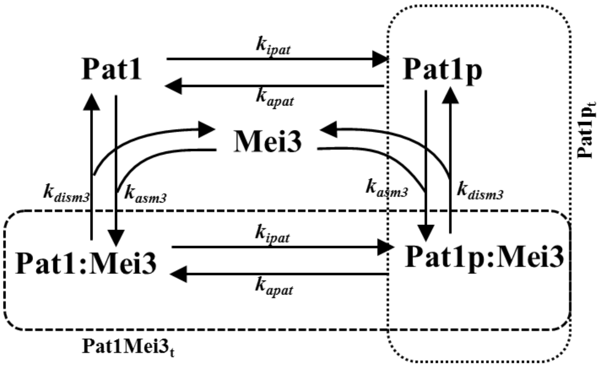

$Pat1_T$ = *Pat1* + *Pat1p* + *Pat1:Mei3* + *Pat1p:Mei3*
$Pat1Mei3_t$ = *Pat1:Mei3* + *Pat1p:Mei3*
$Pat1_{pt}$ = *Pat1p* + *Pat1p:Mei3* (Total PheS inhibited Pat1 forms)

$$\frac{dPat1_{pt}}{dt} = k_{ipat} . \ PheS. \ \left(Pat1_T - Pat1_{pt}\right) - k_{apat}. Pat1_{pt}$$

$$\frac{dPat1Mei3_t}{dt} = k_{asm3} . \ (Pat1_T - Pat1Mei3_t) . (Mei3_T - Pat1Mei3_t) - (k_{dism3} + \ k_{dmei3}). Pat1Mei3_t$$

The active form of Pat1 is calculated using the algebraic expression:

$$Pat1 = \left(1 - \frac{Pat1Mei3_t}{Pat1_T}\right) . (Pat1_T - \ Pat1_{pt})$$

XPPAUT code consisting of equations and parameter values for Ste11-Pat1-Mei2 subsystem is provided in the section 1.

Model of mitosis to meiosis transition:

We extended the recent model proposed by Chica *et al* (2016) for the fission yeast cell cycle by integrating the meiosis-specific regulation. The core cell cycle consists of regulation of essential Cdk1:Cdc13 (Cdk1:Cyclin B) complex. Cdk1 is controlled by the (a) synthesis of Cdc13 (which is proportional to the cell mass), (b) stoichiometric inhibitor Rum1 in G1, (c)

inhibitory phosphorylation of Cdk1 by Wee1 kinase and dephosphorylation of it by phosphatase Cdc25 in G2 and (d) APC/C dependent degradation (activated by Cdk1) of Cdc13 during the metaphase to anaphase transition **(Figure 1b)**. In turn, Cdk1 phosphorylates Rum1 and targets it for ubiquitin dependent degradation forming a double negative feedback loop. Further, Cdk1 phosphorylates Wee1 and Cd25 to form double negative and positive feedback loops, respectively. All synthesis, degradation, binding and dissociation reactions are approximated by the law of mass action. Reversible phosphorylation/dephosphorylation reactions (of Cdc25, Wee1, IE and APC) are described by Michaelis Menten kinetics.

In addition to regulation of Cdk1:Cdc13, the model includes the regulation of phosphatase PP2A:B55, which controls the G2/M transition by activating Wee1 and inhibiting Cdc25. Ppk18 (Gw) phosphorylated Igo1 inhibits PP2A:B55 by forming inhibitory complex.

$$\mathbf{Igo1_p + PP2A} \underset{k_{d55}}{\overset{k_{a55}}{\rightleftharpoons}} \mathbf{Igo1_p{:}PP2A} \xrightarrow{k_{pp2a}} \mathbf{Igo1 + PP2A}$$

$$Igo1p{:}PP2A = \frac{\beta - \sqrt{(\beta^2 - 4\,.\,Igo1_{pt}\,.\,PP2A_t)}}{2}$$

$$\beta = Igo1_{pt} + PP2A_t + (k_{d55} + k_{pp2a})/k_{a55}$$
$$PP2A_t = PP2A + Igo1_p{:}PP2A$$

Gw is activated by Cdk1-dependent phosphorylation. We considered that the Tor2/PKA activity controls (i) Gw activation by Cdk1(k'_{imu}), (ii) the growth rate of the fission yeast (mu_{max}) and (iii) the utilization of internal nitrogen sources for the Cdc13 synthesis under nitrogen starvation (k''_{scyc}). Further, we considered that Cdk1 controls Ste11 binding to its promoter (increases k_{mste11}) and in turn Ste11 inhibits Cdk1 via PheS, which promotes Rum1 accumulation (PheS decreases the Cdk1 dependent degradation rate of Rum1, k''_{dcki}). *XPPAUT* code consisting of equations and parameter values for the integrated model is provided in the section 2.

Section 1: XPPAUT code for the Mei2-Ste11-Pat1 subsystem

```
# XPPAUT code that can be copied to a text file with *.ode extension
# Bifurcation diagrams can be obtained by using kimste11 as a bifurcation parameter

#Initial conditions for dynamic variables
initial STE11T=0.477, STE11N=0.034, RPOL=0.246, PHES=0.026, MEI3T=0, PAT1PT=0.013, PAT1MEI3T=0, MEI2T=0.101, MEI2PT=0.096, MEI2TT=0.048, MEI2PP=0.0455

# Values of kinetic parameter
par Pat1T=1, Tor2=0, PKA=0.75
par ksste11=0.2, ksste11'=0.1, kdste11=0.05, kmste11=0.01, krst=0.025
par kexste11m=0, kexste11=1, kexste11'=4, kexste11"=2, kimste11=0.5, kimste11'=1.5
par kapol=0.014, kapol'=0.1, kipol=0.06
par ksphe=0.1, kdphe=0.05, kmphe=0.3
par ksmatm=0.06, kdmatm=0.04, kmmat=0.4
par ksmei3=0.03, kdmei3=0.02
par kipat=0.1, kapat=0.2
par kasm3=100, kdism3=0.1
par ksmei2=0, ksmei2'=0.007, kdmei2=0.005, kdmei2'=0.01, kmei2=0.1
par kpmei2=1, kdpmei2=0.2, Jpmei2=0.03
par kpmei2'=0.1, kdpmei2'=0.1, Jtmei2=1
par hs=2, hp=2, he=2, hm=5


# Set of differential equations:
# Ste11t represents the total concentration of Ste11
Ste11t' = (ksste11*(krst/(krst + PKA)) + ksste11'*Ste11n^hs/(kmste11^hs + Ste11n^hs))*Rpol - kdste11*Ste11t

# Ste11n represents the nuclear concentration of Ste11
Ste11n' = (kimste11 + kimste11'*PheS)*(Ste11t - Ste11n) - (kexste11m + kexste11*mu + kexste11'*Pat1 + kexste11"*mu*Pat1)*Ste11n - kdste11*Ste11n

# Rpol represents the RNA polymerase II (Pol II CTD) activation by Mei2
Rpol' = (kapol + kapol'*(Mei2T-Mei2pp))*(1 - Rpol) - kipol*Rpol

# PheS represents the upregulation of pheromone signalling pathway
PheS' = ksphe*Ste11n^hp/(kmphe^hp + Ste11n^hp)- kdphe*PheS

# Mei3T represents total concentration of stoichiometric inhibitor (free and complex with Pat1)
Mei3T' = ksmei3*Matpm - kdmei3*Mei3T

# Pat1pt is the total concentration of inactive Pat1 (free and in complex with Mei3)
Pat1pt' = kipat*PheS*(Pat1T - Pat1pt) - kapat*Pat1pt


# Pat1Mei3t represents the total concentration of complexes between Pat1 and Mei3
```

```
Pat1Mei3t'= kasm3*(Pat1T-Pat1Mei3t)*(Mei3T-Pat1Mei3t) - (kdism3+kdmei3)*Pat1Mei3t

# Mei2T is total concentration of Mei2
Mei2T' = ksmei2 + ksmei2'*Ste11n^he/(Ste11n^he + kmei2^he) - kdmei2*(Mei2T - Mei2pp) - kdmei2'*Mei2pp

# Mei2pt represents total concentration of Pat1 phosphorylated forms
Mei2pt' = kpmei2*Pat1*(Mei2T - Mei2pt)/(Mei2T - Mei2pt + Jpmei2) - kdpmei2*Mei2pt/(Mei2pt + Jpmei2) - kdmei2*(Mei2pt - Mei2pp)  - kdmei2'*Mei2pp

# Mei2tt represents total concentration of Tor2 phosphorylated forms
Mei2tt' = kpmei2'*Tor2*(Mei2T - Mei2tt)/(Mei2T - Mei2tt + Jtmei2) - kdpmei2'*Mei2tt/(Mei2tt + Jtmei2)  - kdmei2*(Mei2tt - Mei2pp) - kdmei2'*Mei2pp

# Mei2pp represents Tor2 and Pat1 phosphorylated forms
Mei2pp' = kpmei2'*Tor2*(Mei2T - Mei2tt - Mei2)/(Mei2T - Mei2tt + Jtmei2) - kdpmei2'*Mei2pp/(Mei2tt + Jtmei2) + kpmei2*Pat1*(Mei2T-Mei2pt-Mei2)/(Mei2T-Mei2pt + Jpmei2) - kdpmei2*Mei2pp/(Mei2pt + Jpmei2) - kdmei2'*Mei2pp

# Set of algebraic expression:
# Pat1 is active form of the kinase
Pat1 = (1- (Pat1Mei3T/Pat1T))*(Pat1T - Pat1pt)
aux Pat1 = Pat1

# Mat1-Pm gene expression
Matpm = (ksmatm/kdmatm)*ste11n^hm/(kmmat^hm + ste11n^hm)
aux Matpm = Matpm

# Mei2a is the meiosis activator
Mei2 = Mei2T - Mei2tt - (Mei2pt - Mei2pp)
Mei2a = Mei2T - Mei2pt
aux Mei2a = Mei2a

# Ste11c is cytoplasmic concentration of Ste11
Ste11c = Ste11t - Ste11n
aux Ste11c = Ste11t - Ste11n

# Growth kinases
mu = PKA*Tor2

@ total=400,dt=0.1,meth=STIFF
@ xlo=0,xhi=400,ylo=0,yhi=2.5
@ NTST=15,NMAX=20000000,NPR=1000000,DS=0.02
@ DSMAX=0.01,DSMIN=0.001,PARMIN=-10,PARMAX=0.6
@ AUTOXMIN=0,AUTOXMAX=1,AUTOYMIN=0,AUTOYMAX=1.8
@ NPLOT=8, yp1=Ste11t, yp2=PheS, yp3=Mei2a, yp4=Pat1, yp5=Ste11n, yp6=Mei2T, yp7=Mei3T, yp8=Matpm

done
```

Section 2: XPPAUT code for the integrated model of mitosis to meiosis transition

```
# XPPAUT code that can be copied to a text file with *.ode extention
# Nutrient Rich: Tor2=1, PKA=1
# Nutrient Starvation: Tor2=0, PKA=0.75

# The core fission yeast cell cycle model
# Initial conditions for dynamic variables
initial CycBT=0.681, Cdk1=0.112, pCdk1=0.559, CKIT=0.011, Complex=0.0015,
Wee1=0.99, Cdc25P=0.009, IE=0.008, APC=0, M=0.815, GWi=0.926, GWP=0.04,
Igo1pT=0.183

# Values of kinetic parameter
par tor2=0, PKA=0.75
par kscyc=0.05, kscyc"=0.005, kdcyc'=0.05, kdcyc"=0.25, kdcyc'"=0.5, kgri=0.05, kaie=0.1,
kiie=0.025, Jie=0.01, kaapc=0.5, kiapc=0.125, Japc=0.01
par kscki=0.1, kdcki'=0.3, kdcki"=80, kass=100, kdiss=0.1, mumax=0.0046, thres=0.2
par kwee'=0.1, kwee"=1, Vawee'=0.1, Vawee"=0.15, Viwee=1, Jwee=0.01
par k25'=0.1, k25"=4, Va25=1, Vi25'=0.1, Vi25"=0.15, J25=0.01
par GWtotal=1, kamu=0.04, kimu=0, kimu'=1, kgwa=1, kgwi=0.1
par Igo1t=5, PP2At=1, kIgo1p=1, kpp2a=1, ka55=100, kd55=1

# Set of differential equations
# CycBT represents the sum of all Cdk1:Cdc13 forms
CycBT' = kscyc*M + kscyc"*kgri/(kgri + Tor2) - (kdcyc' + kdcyc'"*APC)*CycBT -
kdcyc"*(Complex + Complex2)

# Cdk1 and pCdk1 represent unphosphorylated and phosphorylated Cdc2:Cdc13
complexes, respectively
Cdk1' = kscyc*M + kscyc"*kgri/(kgri + Tor2) -   kwee*Cdk1 + k25*pCdk1 -   (kdcyc' +
kdcyc'"*APC)*Cdk1  -   kass*Cdk1*CKI + (kdiss + Vdcki)*Complex
pCdk1' = kwee*Cdk1 -   k25*pCdk1 -   (kdcyc' + kdcyc'"*APC)*pCdk1  -   kass*pCdk1*CKI
+ (kdiss + Vdcki)*Complex2

# CKIT represents the total level of stoichiometric Cdk inhibitor, Rum1
CKIT'  = kscki - Vdcki*CKIT

# Complex and Complex2 correspond to unphosphorylated and Y-
phosphorylated Cdc2:Cdc13:Rum1 trimers, respectively
Complex' = - kwee*Complex + k25*Complex2 - (kdcyc' + kdcyc'"*APC +
kdcyc")*Complex  + kass*Cdk1*CKI - (kdiss + Vdcki)*Complex
Complex2 = CycBT - Cdk1 - pCdk1 - Complex
CKI = CKIT - Complex - Complex2

# Wee1 and Cdc25P are the active forms of the tyrosine--modifying enzymes
(Wee1/Mik1  and Cdc25)
Wee1' = (Vawee' + Vawee"*PP2A)*(1 - Wee1)/(Jwee + 1 - Wee1) -
Viwee*Cdk1*Wee1/(Jwee + Wee1)
Cdc25P' = Va25*Cdk1*(1  - Cdc25P)/(J25  + 1 - Cdc25P) - (Vi25' +
Vi25"*PP2A)*Cdc25P/(J25  + Cdc25P)
```

```
# APC/C is activated by Cdc2:Cdc13 complex indirectly (through IE)
IE' = kaie*(1-IE)*Cdk1/(Jie + 1 - IE) - kiie*IE/(Jie + IE)
APC' = kaapc*IE*(1-APC)/(Japc + 1 - APC) - kiapc*APC/(Japc + APC)

# exponential growth of cell mass is assumed with minimal cycle time of 150
mins (mumax=0.0046)
M'  = mumax*mu*M

# the cell divides when the active Cdk1:Cdc13 complex drops below a threshold value (thres)
global -1 {Cdk1-thres} {M=0.5*M}

# Rate functions
Vdcki = kdcki' + kdcki"*Cdk1*1/(1+PheS)
kwee   = kwee' + (kwee" - kwee')*Wee1
k25    = k25' + (k25" - k25')*Cdc25P

# The model for the growth rate regulated Greatwall/Endosulfine/PP2A pathway
# Gwi represents the form of Gw inactivated by PKA and TOR kinases
Gwi' = (kimu + kimu'*mu)*(GWtotal - Gwi - Gwp) - kamu*Gwi

# Gwp is the Cdk1:Cdc13 activated form of Greatwall-kinase
Gwp' = kgwa*Cdk1*(GWtotal - Gwi - Gwp) - kgwi*Gwp

# Igo1 (Igo1) is phosphorylated by Gwp and dephosphorylated by PP2A
Igo1pt' = kIgo1p*Gwp*(Igo1t - Igo1pt) - kpp2a*Igo1pPP2A

# The level of Igo1pPP2A complex (Igo1p:PP2A) is assumed to be in pseudo-steady state
BB = Igo1pt + PP2At + (kd55 + kpp2a)/ka55
Igo1pPP2A = (BB -  sqrt(BB^2 -  4*Igo1pt*PP2At))/2
PP2A = PP2At - Igo1pPP2A
aux PP2A = PP2At - Igo1pPP2A

# Relative growth rate (mu) has values between 0 and 1 (rich medium: mu=1,
Nitrogen starvation: mu=0).
# Starvation can induce growth arrest in Tor2/PKA dependent and independent manner.
mu = PKA*Tor2

##############################################################################
# Mei2-Ste11-Pat1 subsystem
# Initial conditions for dynamic variables
initial STE11T=0.0321, STE11N=0.003, RPOL=0.197, PHES=0, MEI3T=0, PAT1PT=0,
PAT1MEI3T=0, MEI2T=0.00237, MEI2PT=0.00199, MEI2TT=0.00107, MEI2PP=0.0008

# Values of kinetic parameter
par Pat1T=1
par ksste11=0.2, ksste11'=0.1, kdste11=0.05, kmste11=0.003, krst=0.025
par kexste11m=0, kexste11=1, kexste11'=4, kexste11"=2, kimste11=0.5, kimste11'=1.5,
kicdk=0.01
par kapol=0.014, kapol'=0.1, kipol=0.06
```

```
par ksphe=0.1, kdphe=0.05, kmphe=0.3
par ksmatm=0.06, kdmatm=0.04, kmmat=0.4
par ksmei3=0.03, kdmei3=0.02
par kipat=0.1, kapat=0.2
par kasm3=100, kdism3=0.1
par ksmei2=0, ksmei2'=0.007, kdmei2=0.005, kdmei2'=0.01, kmei2=0.1
par kpmei2=1, kdpmei2=0.2, Jpmei2=0.03
par kpmei2'=0.1, kdpmei2'=0.1, Jtmei2=1
par hs=2, hp=2, he=2, hm=5

# Set of differential equations
# Ste11t represents the total concentration of Ste11
Ste11t' = (ksste11*(krst/(krst + PKA)) + ksste11'*Ste11n^hs/(kmste11c^hs +
Ste11n^hs))*Rpol - kdste11*Ste11t

# Ste11n represents the nuclear concentration of Ste11
Ste11n' = (kimste11 + kimste11'*PheS)*(Ste11t - Ste11n) - (kexste11m + kexste11*mu +
kexste11'*Pat1 + kexste11''*mu*Pat1)*Ste11n - kdste11*Ste11n

# Rpol represents the RNA polymerase II (Pol II CTD) activation
Rpol' = (kapol + kapol'*(Mei2T-Mei2pp))*(1 - Rpol) - kipol*Rpol

# PheS represents the upregulation of pheromone signalling pathway
PheS' = ksphe*Ste11n^hp/(kmphe^hp + Ste11n^hp)- kdphe*PheS

# Mei3T represents total concentration of stochiometric inhibitor (free and complex with
Pat1)
Mei3T' = ksmei3*Matpm - kdmei3*Mei3T

# Pat1pt is total concentration of inactive Pat1 (free and in complex with Mei3)
Pat1pt' = kipat*PheS*(Pat1T - Pat1pt) - kapat*Pat1pt

# Pat1Mei3t represents the total concentration of complexes between Pat1 and Mei3
Pat1Mei3t'= kasm3*(Pat1T-Pat1Mei3t)*(Mei3T-Pat1Mei3t) - (kdism3+kdmei3)*Pat1Mei3t

# Mei2T is total concentration of Mei2
Mei2T' = ksmei2 + ksmei2'*Ste11n^he/(Ste11n^he + kmei2^he) - kdmei2*(Mei2T - Mei2pp)
- kdmei2'*Mei2pp

# Mei2pt represents total concentration of Pat1 phosphorylated forms
Mei2pt' = kpmei2*Pat1*(Mei2T - Mei2pt)/(Mei2T - Mei2pt + Jpmei2) -
kdpmei2*Mei2pt/(Mei2pt + Jpmei2) - kdmei2*(Mei2pt - Mei2pp)  - kdmei2'*Mei2pp

# Mei2tt represents total concentration of Tor2 phosphorylated forms
Mei2tt' = kpmei2'*Tor2*(Mei2T - Mei2tt)/(Mei2T - Mei2tt + Jtmei2) -
kdpmei2'*Mei2tt/(Mei2tt + Jtmei2)  - kdmei2*(Mei2tt - Mei2pp) - kdmei2'*Mei2pp

# Mei2pp represents Tor2 and Pat1 phosphorylated forms
```

```
Mei2pp' = kpmei2'*Tor2*(Mei2T - Mei2tt - Mei2)/(Mei2T - Mei2tt + Jtmei2) -
kdpmei2'*Mei2pp/(Mei2tt + Jtmei2) + kpmei2*Pat1*(Mei2T-Mei2pt-Mei2)/(Mei2T-Mei2pt
+ Jpmei2) - kdpmei2*Mei2pp/(Mei2pt + Jpmei2) - kdmei2'*Mei2pp

# Set of algebraic expression
# Pat1 is active form of the kinase
Pat1 = (1- (Pat1Mei3T/Pat1T))*(Pat1T - Pat1pt)
aux Pat1 = Pat1

# Mat1-Pm gene expression
Matpm = (ksmatm/kdmatm)*ste11n^hm/(kmmat^hm + ste11n^hm)
aux Matpm = Matpm

# Mei2a is the meiosis activator
Mei2 = Mei2T - Mei2tt - (Mei2pt - Mei2pp)
Mei2a = Mei2T - Mei2pt
aux Mei2a = Mei2a

# Ste11c is cytoplasmic concentration of Ste11
Ste11c = Ste11t - Ste11n
aux Ste11c = Ste11t - Ste11n

# Regulation of Ste11 binding by Cdk1
kmste11c = kmste11*(1 + Cdk1/kicdk)

@ total=500,dt=0.1,meth=STIFF
@ xlo=0,xhi=500,ylo=0,yhi=2.5
@ NTST=15,NMAX=20000000,NPR=1000000,DS=0.02
@ DSMAX=0.01,DSMIN=0.001,PARMIN=-10,PARMAX=0.6
@ AUTOXMIN=0,AUTOXMAX=1,AUTOYMIN=0,AUTOYMAX=1.8
@ NPLOT=8, yp1=M, yp2=Cdk1, yp3=CycBT, yp4=CKIT, yp5=Ste11t, yp6=Mei2a,
yp7=Pat1, yp8=PheS

done
```

Supplementary table and figures:

Table S1: Parameter values used to simulate various experimental situations.

Nitrogen Starvation		Nitrogen Rich	
**Genotype**	**Parametric changes**	**Genotype**	**Parametric changes**
rst2Δ	k_{rst}=0	*pka1Δ/ cyr1Δ*	PKA = 0
mei2Δ	k'_{smei2}=0	*tor2-51(32°C)*	Tor2=0
mei3Δ	k_{smei3}=0	*tor2-51cgs1Δ*	Tor2=0, PKA=5
mat1pmΔ	k_{smat}=0	*tor2-51pka1Δ*	Tor2=0, PKA=0
spk1Δ	k_{sphe}=0	*ste11 overexpression*	k'_{sste11}=0.2
ste11Δ	k_{sste11}=k'_{sste11}=0	*ste11^{T82A}*	k_{mste11}=0.001
ste11$^{T173D, S218D}$	$k_{exste11m}$=5	*ste11$^{T173A, S218A}$*	$k'_{exste11}$=3.2
ste11$^{T305A, T317A}$	$k'_{imste11}$=0.15	*WT in leptomycin B*	$k_{exste11}$=0.1, $k'_{exste11}$=0.4, $k''_{exste11}$=0.2
ste11^{T82D}	k_{mste11}=0.1	*pat1-114 at 30°C*	$Pat1_T$=0.5
lsk1Δ	k'_{apol}=0	*ste11$^{T305D, T317D}$*	$k_{imste11}$=2
nmt-tor2	Tor2=5, k_{dmei2}=0.02	*mts2Δ*	k_{dmei2}=0.0005, k'_{dmei2}=0.001
tor2-s65	Tor2=1	*tor2-ts6 mei2Δ*	Tor2=k'_{smei2}=0
tor2-s65 pREP41-mei2	Tor2=1, k_{smei2}=0.004	*pat1-114 (34°C)*	$Pat1_T$=0.001
nmt-tor2 cyr1Δ	Tor2=5, k_{dmei2}=0.02, PKA=0	*mei2Δ pat1-114(34°C)*	k'_{smei2}=0, $Pat1_T$=0.001
		nmt-tor2 pat1-114 (34°C)	Tor2=5, k_{dmei2}=0.02, $Pat1_T$=0.001
		ste11Δ pat1-114 (34°C)	k_{sste11}=k'_{sste11}=0, $Pat1_T$=0.001
		lsk1Δ pat1-114 (34°C)	k'_{apol}=0, $Pat1_T$ =0.001
		mei2-SATA	k_{pmei2}=0
		mei2-8A	k'_{pmei2}=0
		mei2-8A-SATA	k_{pmei2}=k'_{pmei2}=0
		lsk1Δ mei2-L-SATA	k'_{apol}=k_{pmei2}=0
		nmt1-ste11 lsk1Δ	k'_{sste11}=0.2, k'_{apol}=0
		*pat1Δ*mei2Δ*nmt1-mei2*	k'_{smei2}=0, k_{smei2}=0.004, Pat1T=0.001
		mei2Δnmt1-mei2	k'_{smei2}=0, k_{smei2}=0.004
		byr2-ΔN	k_{sphe}=0.4
		byr2-ΔN mei3Δ	k_{sphe}=0.4, ksmei3=0

* Heterothallic mutational situations are simulated with k_{smei3}=0

**Modelling the experimental situations given below suggest that PKA is not completely inactivated under nitrogen starvation or its inactivation under nitrogen starvation depends on Tor2 activity.

1. *nmt-tor2* (overexpression) + nitrogen starvation = blocks sexual differentiation.
2. *tor2-s65* (active, sterile mutant) + nitrogen starvation = Block sexual differentiation.
3. *cyr1Δ* + *nmt-tor2* (overexpression) + nitrogen starvation = sexual differentiation occurs because PKA is inactive completely.
4. *tor2*-51(Tor2 inactivation) = mating in rich medium.
5. *tor2-51* + *cgs1Δ* = block sexual differentiation. PKA is hyperactive.

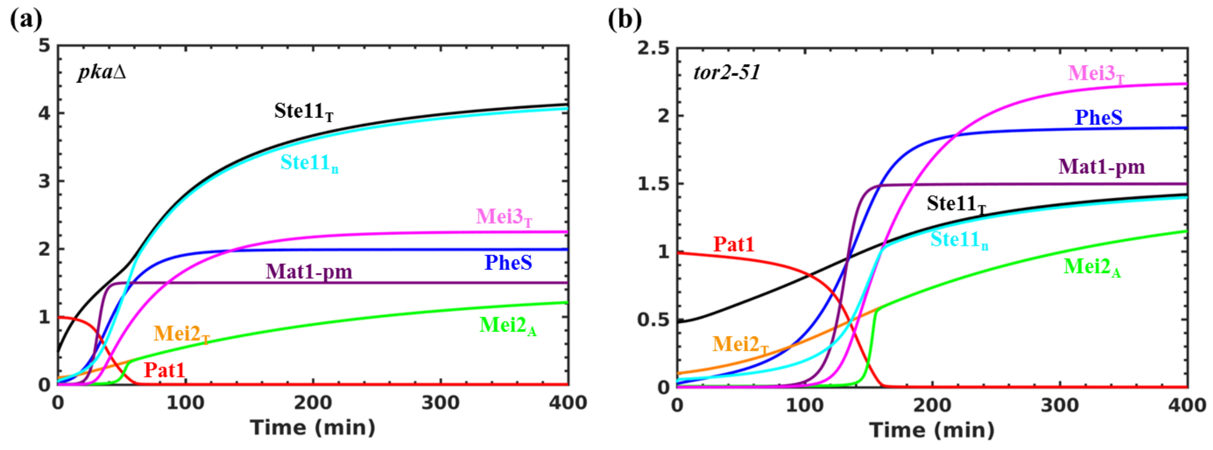

Figure S1: Effect of either PKA or Tor2 inactivation. **(a)** PKA =0, (**b**) Tor2=0

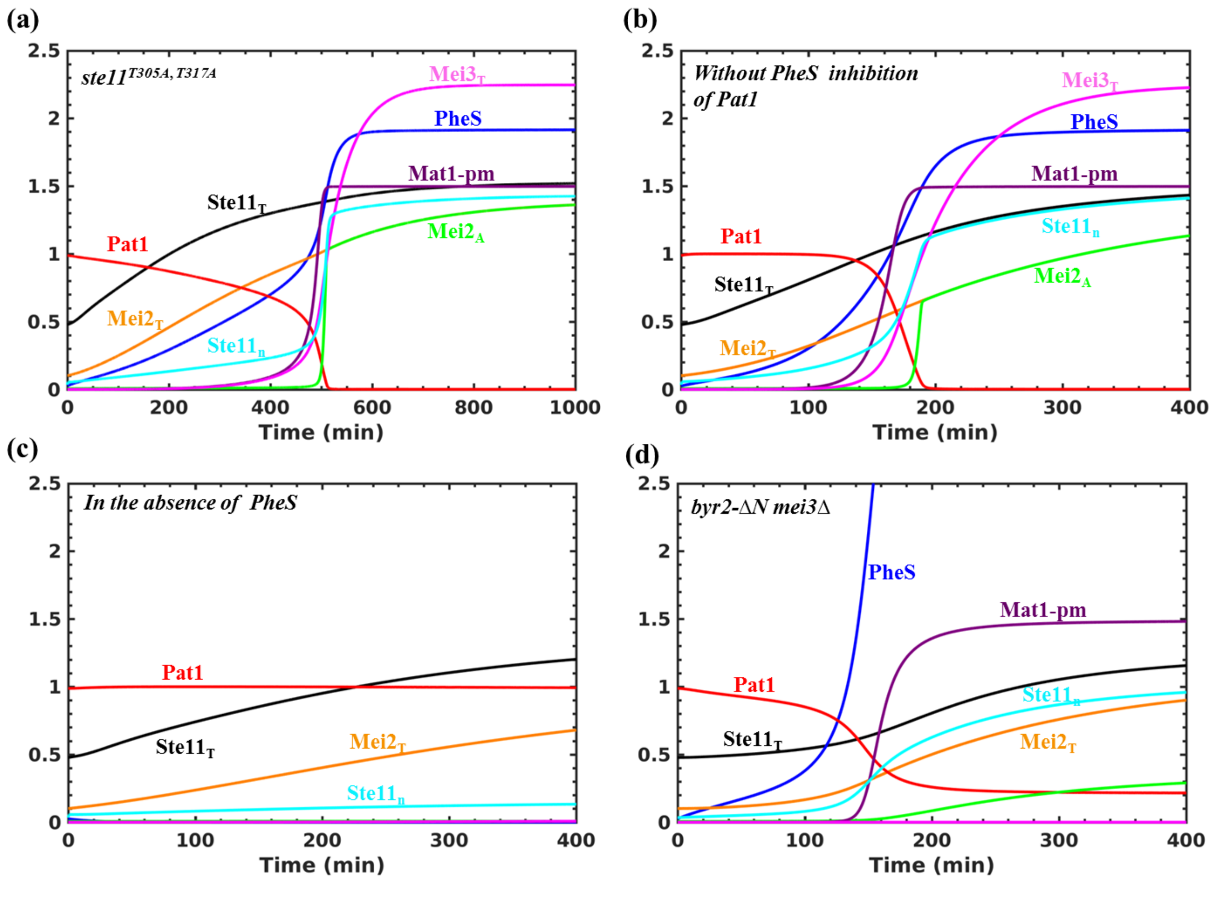

Figure S2: PheS-dependent regulation of conjugation and meiosis. Nitrogen starvation induced meiotic entry is unaffected (**a**) in the absence of PheS dependent regulation of Ste11 import ($k_{imste11}$,=0.15) and **(b)** in the absence of Pat1 inhibition by PheS (k_{ipat}=0) but is affected (**c**) in the absence of PheS (k_{sphe}=0). **(d)** Hyperactive PheS signalling leads to ectopic meiosis in nutrient rich conditions and is Mei3 independent (k_{sphe}=0.4, k_{smei3}=0).

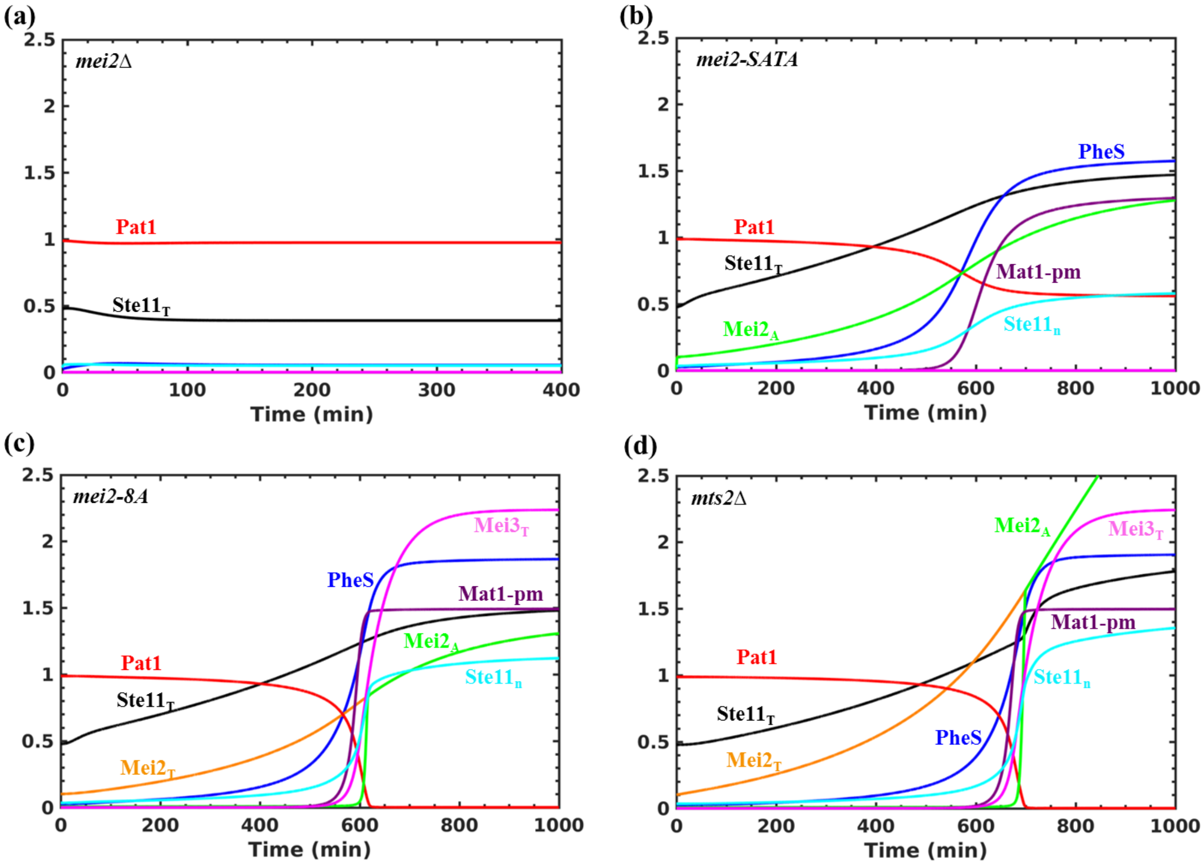

Figure S3: Mei2-dependent regulation of conjugation and meiosis**. (a)** Under nitrogen starvation, Mei2 is required for the conjugation and meiosis (k'_{smei2}=0, Tor2=0, PKA=0.75). Under nitrogen rich conditions (Tor2=1, PKA=1), the meiotic entry is induced in the absence of **(b)** Mei2 phosphorylation by Pat1 (k_{pmei2}=0, k_{smei3}=0) and **(c)** Mei2 phosphorylation by Tor2 ($k_{pmei2'}$=0), and **(d)** in the absence of degradation of Mei2 (k_{dmei2}=0.0005, $k_{dmei2'}$=0.001).

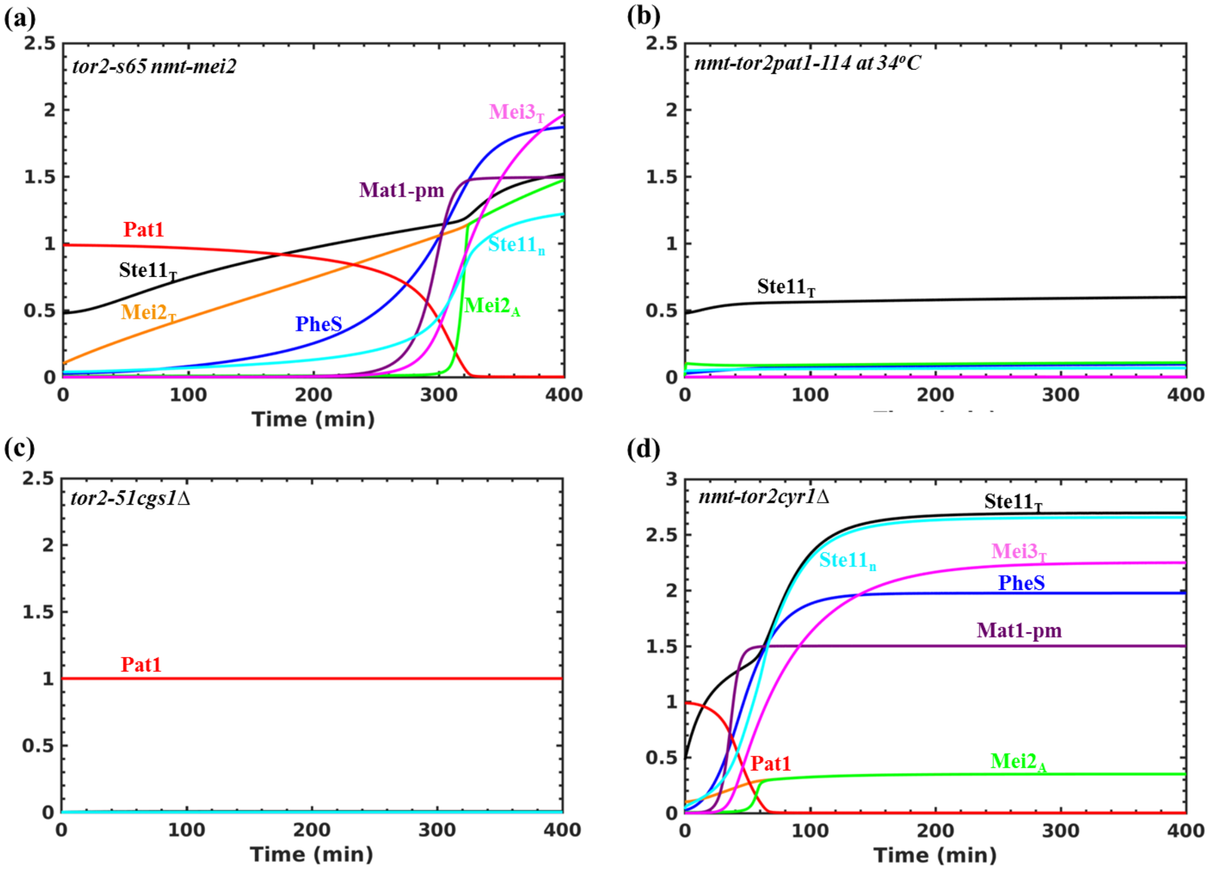

Figure S4: Effect of Tor2 and PKA on meiotic entry. **(a)** Tor2 sterile mutant phenotype is suppressed by Mei2 overexpression (Tor2=1, k_{smei2}=0.004). **(b)** *pat1-114* induced ectopic meiosis is suppressed by Tor2 overexpression (Tor2=5, k_{dmei2}=0.02, $Pat1_T$=0.001). **(c)** Tor2 inactivated meiosis is suppressed by PKA hyper-activation (Tor2=0, PKA=5). **(d)** The inhibition on conjugation and meiosis by Tor2 overexpression is suppressed by PKA inactivation (Tor2=5, k_{dmei2}=0.02, PKA=0).

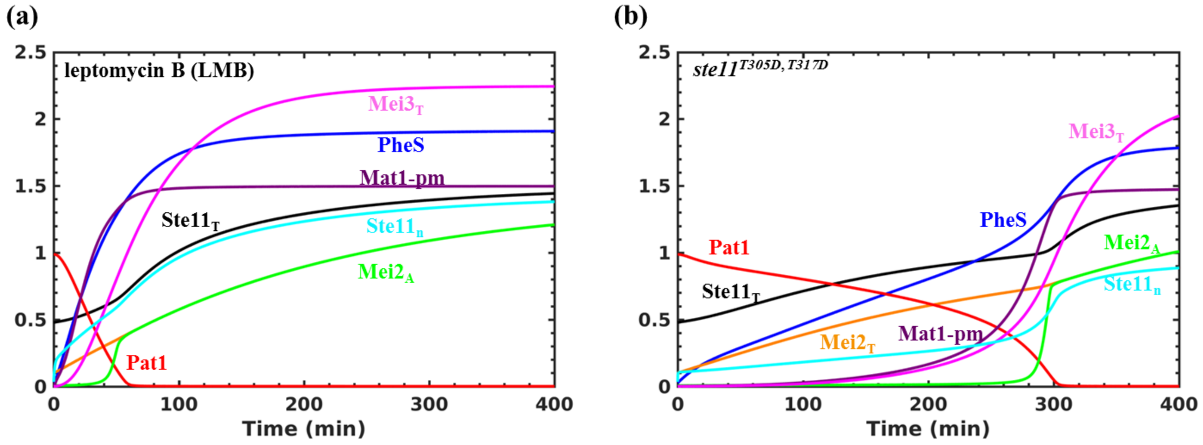

Figure S5: Sensitivity of the model to the nuclear import and export rate of Ste11. Under nitrogen rich conditions, **(a)** reducing the nuclear export rate ($k_{exste11}$=0.1, $k_{exste11'}$=0.4, $k_{exste11''}$=0.2) or **(b)** increasing the nuclear import rate ($k_{imste11}$=2) leads to meiotic entry.
